# Tuning collagen nonlinear mechanics with interpenetrating networks drives adaptive cellular phenotypes in three dimensions

**DOI:** 10.1126/sciadv.adt3352

**Published:** 2025-06-20

**Authors:** Marco A. Enriquez Martinez, Zhao Wang, Yanina D. Alvarez, Jade E. O’Neill, Robert J. Ju, Petri Turunen, Melanie D. White, Jitendra Mata, Elliot P. Gilbert, Jan Lauko, Alan E. Rowan, Samantha J. Stehbens

**Affiliations:** ^1^ Australian Institute for Bioengineering and Nanotechnology (AIBN), The University of Queensland, Brisbane, QLD 4072, Australia.; ^2^ Institute for Molecular Biosciences (IMB), The University of Queensland, Brisbane, QLD 4072, Australia.; ^3^ Institute of Molecular Biology, Mainz 55128, Germany.; ^4^ School of Biomedical Sciences (SBMS), The University of Queensland, Brisbane, QLD 4072, Australia.; ^5^ Australian Centre for Neutron Scattering, Australian Nuclear Science and Technology Organisation (ANSTO), Lucas Heights, NSW 2234, Australia.; ^6^ School of Chemistry, University of New South Wales, Sydney, NSW 2052, Australia.

## Abstract

In living tissues, collagen networks rarely exist alone because they are embedded within other biological matrices. When combined, collagen networks rigidify via synergistic mechanical interactions and stiffen only with higher mechanical loads. However, how cells respond to the nonlinear elasticity of collagen in hybrid networks remains largely unknown. Here, we demonstrate that when collagen rigidifies by the interpenetration of a second polymer, the amount of force that initially stiffens the network (onset of stiffening, σ_c_) increases and is sufficient to stimulate an increase in intracellular tension. We investigated this effect by precisely controlling the nonlinear elasticity of collagen with the synthetic semiflexible polymer, polyisocyanopeptides. We find that small increases in σ_c_ induce a biphasic response in cell-matrix interactions, influencing how cells migrate, proliferate, and generate contractile force. Our results suggest that cells adaptively respond to changes in the nonlinear mechanics of collagen, which may be a mechanistic behavior used during tissue homeostasis or when collagen rigidifies during pathological conditions.

## Teaser

This work demonstrates that changes in the nonlinear elasticity of collagen in hybrid networks tunes cell-matrix interactions.

## INTRODUCTION

The architecture of mammalian tissues is maintained by the constant secretion and degradation of collagen (*1*). From a mechanical perspective, this constant production of collagen protects tissues such as skin, lungs, and cartilage from tearing when they are stretched or compressed (*2*–*5*). Collagen networks of type I origin, in particular, provide this mechanical resistance to tissues due to their nonlinear response to applied force. Collagen type I networks respond acutely to small deformations by initially bending, but as applied stress increases, their modulus will increase orders of magnitude before they align and rupture (*6*–*9*). At the tissue level, an extensive body of evidence recognizes that the constant stiffening of collagenous tissues is an important part of tissue growth. Mechanical loading by exercise stimulates collagen synthesis (*10*, *11*), while increased tension within collagen fibrils ensures resistance against enzymatic degradation (*12*). Nevertheless, stiffening of collagen also arises from within the interstitial matrix by cellular movement and remodeling. Cells stiffen networks of collagen to generate traction in mesenchymal-based migration (*13*, *14*) and to communicate over long distances (*15*–*17*).

During disease or injury, such as cancer progression and wound healing, new extracellular matrix (ECM) is excessively deposited into the interstitial space at a faster rate than which it is degraded (*18*–*20*). This mechanism is important for the survival and growth of cancer cells to drive metastasis (*21*) and to facilitate the rapid generation of scar tissue during wound closure (*22*). In these scenarios, it is often reported that tissues rigidify characterized by hardening and a decrease in their elasticity (*23*, *24*). This is often due to an increase in the deposition of collagen and intrafibrillar crosslinking between collagen fibrils (*18*, *25*, *26*). However, other alterations in the interstitial matrix that also lead to the rigidification of tissues include the large deposition of fibrin (*27*) and hyaluronic acid (*28*, *29*). Mechanical models of composite systems demonstrate that the presence of these ECM components synergistically influences the ability of collagen fibers to initially bend, requiring higher levels of stress for collagen networks to stiffen with applied load (*30*–*35*). However, an unanswered question within the field of biological soft matter is how these changes in the nonlinear elasticity of collagen actually modulate cellular behavior.

In this study, we investigated biological responses of cancer cells and fibroblasts to changes in the nonlinear elasticity of collagen by developing a tuneable collagen composite system with polyisocyanopeptides (PIC) (*36*). We precisely tailored the nonlinear mechanical behavior of collagen hydrogels with low PIC concentrations and controllable contour lengths. This revealed that cells acutely respond to small changes in the amount of force required to initially bend collagen networks in three dimensions (onset of stiffening$,\sigma_{c}$ ). Our results show that, as collagen slowly rigidifies and loses sensitivity toward stress, cells exhibit morphological and behavioral changes consistent with an increase in cellular tension. We demonstrate that cells exposed to rigid collagen networks have a reduced ability to form cellular extensions, correlating with a decrease in cell migration persistence and a loss in active deformations of the matrix. We further found that these biological responses are concomitant with a biphasic response of integrin-based adhesions to mechanical load. As collagen rigidifies, cells at first productively engage with the matrix, exhibiting adhesions that grow and elongate at the cellular leading edge. However, when we shift the onset of collagen’s stiffening ( $\sigma_{c}$ ) to high levels of stress beyond a threshold, cells fail to deform the network. As a result, the interaction between cells and the collagen network weakens, suggesting that the onset of stiffening ( $\sigma_{c}$ ) modulates the force loading rate within focal adhesions in three-dimensional (3D) collagen networks. Here, we present a novel collagen composite system capable of precisely tuning the nonlinear elasticity of collagen. We also provide new insights into the mechanical parameters that govern physical interactions between cells and collagen matrices. Application of our novel collagen composite system will generate a deeper understanding of how cells adaptively respond to the rigidification of collagen, giving insight to homeostatic tissue maintenance and pathological conditions, such as fibrosis and cancer.

## RESULTS

### PIC tunes the gelation properties of collagen hydrogels with controllable polymer density and contour length

**Fig. 1.**
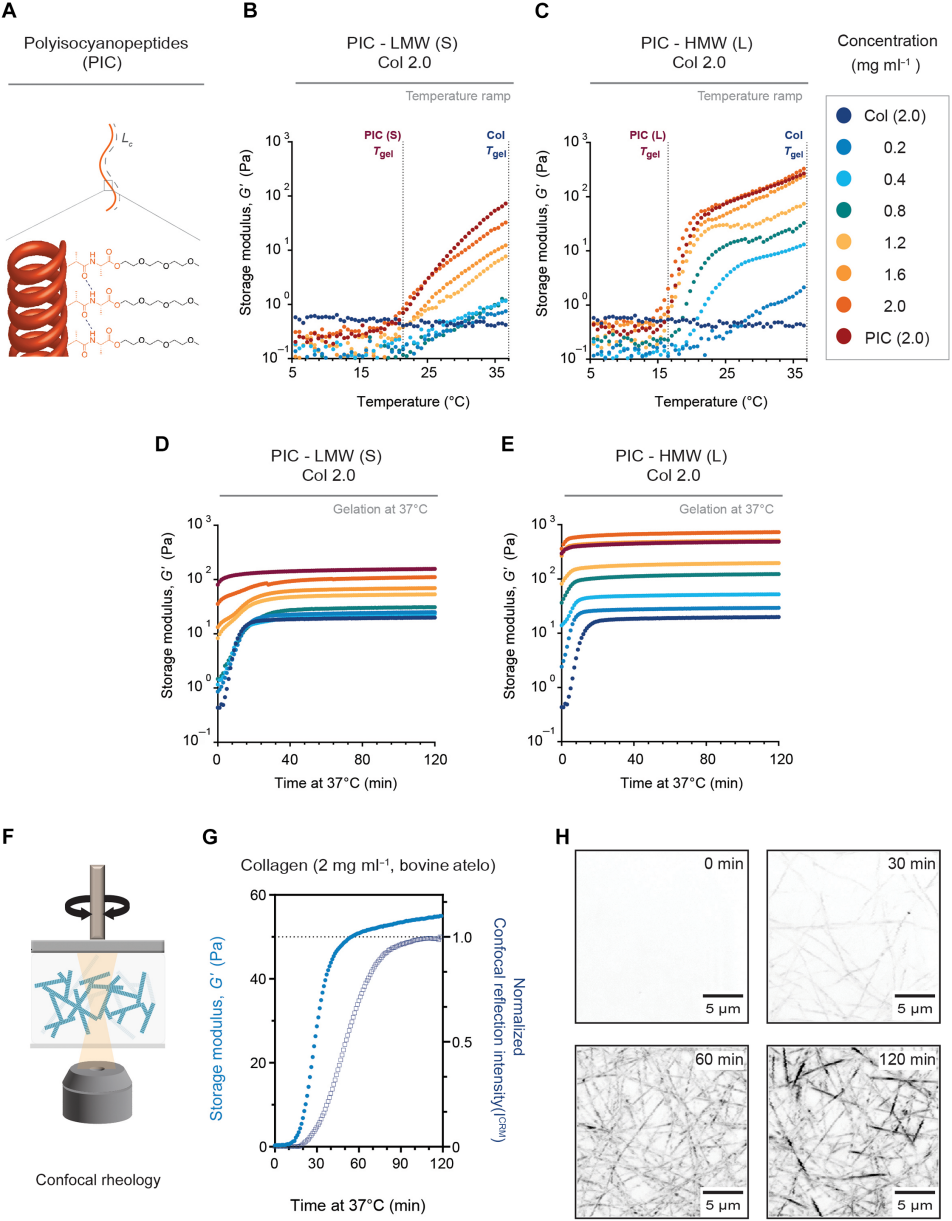
Mechanical properties of PIC-collagen composites. (**A**) Schematic representation of PIC. Contour length *L*_c_ is shown in dotted lines as the end-to-end distance along the polymer backbone. (**B** and **C**) Linear temperature ramps from 5° to 37°C (1°C min^−1^) showing the influence of PIC on the onset of gelation temperature (*T*_gel_) of collagen type I (bovine atelo, 1X PBS, pH 7) of constant concentration (2 mg ml^−1^) with solutions of increasing PIC concentrations (0.2 to 2.0 mg ml^−1^, 1X PBS) and PIC molecular weight (LMW, *M*_v_: 283 kg mol^–1^; and HMW, *M*_v_: 518 kg mol^−1^). (B) LMW (S) PIC and (C) HMW (L) PIC. Gel point (*T*_gel_) defined as the onset in the storage modulus (*G*′, Pa). (**D** and **E**) Gelation profiles of hydrogels incubated in situ for 2 hours at 37°C after temperature ramps. All sets of rheological data are an average of three independent measurements (*n* = 3). (**F**) Schematic of a combined confocal rheology setup. (**G**) Graph shows the evolution of the sol-gel transition [increase in storage modulus (*G*′) (Pa)] at 37°C and the intensity profile in confocal reflection mode (*I*^CRM^) (normalized) of the pure bovine atelo collagen type I [2 mg ml^−1^ and 1X PBS (pH 7)]. Data acquired by simultaneously applying oscillatory shear and acquiring time-lapse images every 1 min. Graph represents data from an average of three independent measurements (*n* = 3). (**H**) Inverted confocal images show the formation of the collagen network every 30 min until the *I*^CRM^ profile reaches a plateau at ~120 min. *Z* projections show 20 μm in depth. Scale bars, 5 μm. The increase in *I*^CRM^ is correlated to the continuing thickening of collagen fibers by lateral growth until the hydrogel undergoes complete gelation.

To control the mechanical behavior of collagen hydrogels, we generated a series of collagen composites where we combined cold solutions of PIC (*36*) with cold solutions of atelo type I collagen before the reconstitution process (Fig. 1A). In this composite system, the collagen concentration was kept constant [2 mg ml^−1^, pH 7, phosphate-buffered saline (PBS), 1X PBS], while the network parameters of PIC were tuned by increasing the PIC density (0.2 to 2.0 mg ml^−1^) or contour length (*L*_c_) of the polymer (low molecular weight (LMW), S; high molecular weight (HMW), L) (*37*, *38*).

To determine the influence of PIC on the mechanical behavior of collagen hydrogels, we first investigated the effect of PIC on collagen’s gelation with oscillatory shear rheology using a temperature ramp (5° to 37°C; at 1°C min^−1^) (Fig. 1, B and C). Our results show that only a minimal addition of the synthetic polymer (0.2 to 0.8 mg ml^−1^) is sufficient to influence the gelation temperature (*T*_gel_) of the collagen-based composites. Consistent with the mechanical profile of PIC (*37*), the *T*_gel_ of the composites is dependent on PIC’s contour length. Collagen composites with LMW PIC (Fig. 1B) gelled at higher temperatures compared to composites with HMW PIC (Fig. 1C; ~22°C versus ~17°C, respectively). Increasing the density of LMW PIC within a concentration range of 0.8 to 2.0 mg ml^−1^ resulted in similar linear moduli (storage modulus, *G′*) to collagen-only hydrogels. The difference in the values of *G′* was small, ranging from ~20 to 100 Pa (Fig. 1D, fig. S1, and table S1). In contrast, HMW PIC composites resulted in moduli ranging from ~20 to 700 Pa (Fig. 1E, fig. S1, and table S2). These rheology experiments demonstrate that PIC can tune the mechanical behavior of collagen hydrogels without altering the gelation temperature or collagen concentration.

### PIC and collagen form an IPN by self-assemblies driven by physiological heat

To ensure that the mechanical enhancement was not due to changes in collagen architecture, we next evaluated the formation of the collagen network in the presence of PIC. Having observed that the composites gelled at lower temperatures, we hypothesized that the formation of the collagen network was accelerated. Previous reports demonstrate that increasing the polymer density of a second network accelerates collagen fibrillogenesis via an effect of macromolecular crowding (MMC) (*39*–*43*). To test this, we first evaluated the formation of atelo type I collagen hydrogels by placing cold collagen solutions between a rheometer and an inverted laser scanning microscope (Fig. 1, F to H). This allowed us to simultaneously monitor the formation of the collagen network structure with changes in its viscoelastic profile as the temperature was increased to 37°C. Atelo collagen hydrogels undergo a phase transition from a solution to a viscoelastic gel (sol-gel transition) at 37°C by showing an increase in storage moduli (*G′*, Pa) accompanied by a delayed increase in reflection intensity [intensity confocal reflectance mode (*I*^CRM^)] (Fig. 1G), indicative of the slow formation of the network structure (Fig. 1H and movie S1).

**Fig. 2.**
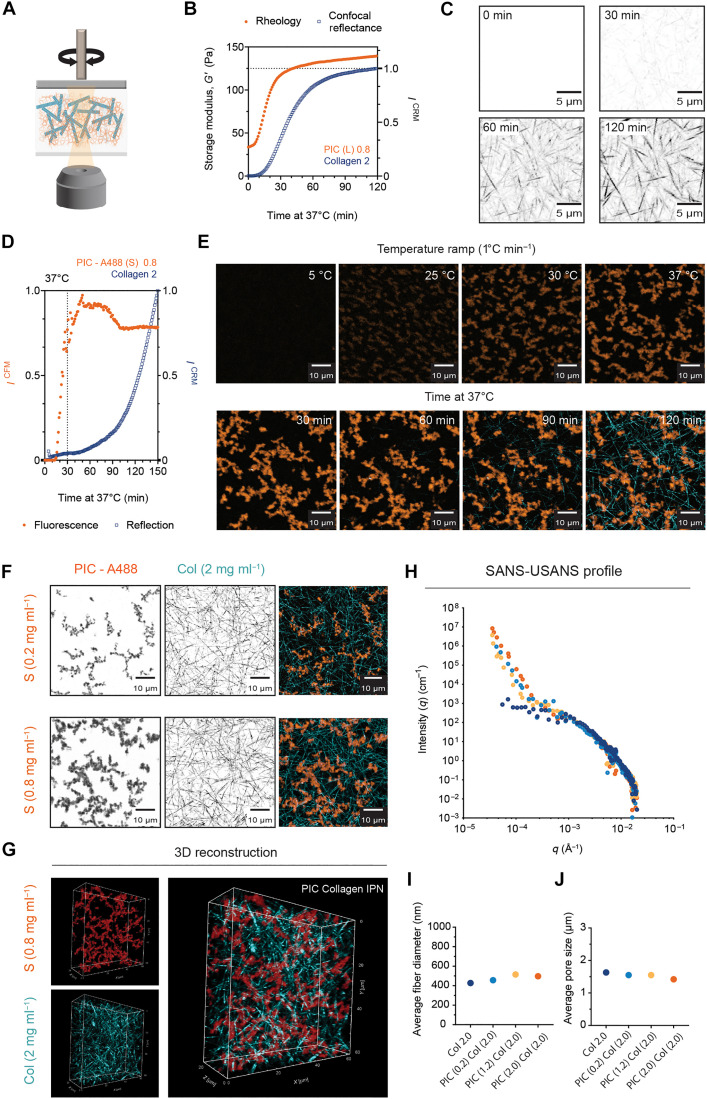
Elucidating collagen’s architecture in PIC-collagen IPNs. (**A**) Schematic of a combined confocal rheology setup. (**B**) Graph shows the gelation of methoxy-functionalized PIC (L, 0.8 mg ml^−1^) with atelo collagen (2 mg ml^−1^). Sets of data are representative from an average of two independent measurements (*n* = 2). (**C**) Inverted confocal images showing the formation of atelo collagen within the composite. *Z* maximum intensity projection (MIP) shows 20 μm in depth. Scale bars, 5 μm. (**D**) PIC-collagen IPN monitored by simultaneous reflectance and fluorescence microscopy. PIC (S, 1:100 azide) fluorescently labeled with DBCO–Alexa 488 [PIC (S)-A488, 1:10 A488/Azide] and atelo collagen monitored by reflection. Quantified reflection and fluorescence intensities obtained from MIPs of 10 μm in depth. Graph shows data from an average of two independent measurements (*n* = 2). (**E**) Gelation from 5° to 37°C (1°C min^−1^) (0 to 30 min) and temperature was kept constant at 37°C for 2 hours (30 to 150 min). Time-lapse images acquired every 1 min. MIPs are 10 μm in depth. Scale bars, 10 μm. (**F**) MIPs of PIC-collagen IPN using PIC-A488 (S, 0.2 mg ml^−1^, 0.8 mg ml^−1^). Images were acquired at 37°C. MIPs show 10 μm in depth. Scale bars, 10 μm. (**G**) 3D reconstruction of a PIC-collagen IPN [PIC (S)–A488, 0.8 mg ml^−1^; collagen, 2.0 mg ml^−1^]. X-Y-Z stack shows a depth of 60 μm by 60 μm by 20 μm. (**H**) Combined USANS-SANS intensity profiles of atelo collagen (2.0 mg ml^−1^) and PIC-collagen composites (S; 0.2, 1.2, and 2.0 mg ml^−1^). Samples were dissolved in 20% D_2_O. Scattering data were fitted and analyzed to determine the influence of PIC in (**I**) collagen’s fiber diameter and (**J**) pore size. SANS/USANS data were obtained from one experiment (*n* = 1).

Next, we monitored the gelation and reflection intensity of collagen composites containing higher concentrations of PIC (Fig. 2, A to C). The lower refractive index of the PIC allowed us to solely monitor the formation of the collagen networks in the absence of any fluorescent labels by time-lapse imaging. In PIC-collagen composites, we observed an immediate increase in *G′* (Pa) at physiological temperature (37°C) (Fig. 2B). The delayed increase in *I*^CRM^ relative to *G′* (Pa) indicated that the reconstitution of the collagen network was not accelerated by the gelation of PIC and that there were no changes in the duration of the reconstitution. Instead, the assembly of the collagen network was only initiated when exposed to physiological temperature (Fig. 2C; 0 to 120 min at 37°C). Higher concentrations of PIC (0.8 to 2.0 mg ml^−1^) showed a similar trend (fig. S2), indicating that the increase in PIC density does not induce an MMC effect.

To visualize the order of network formation, we performed simultaneous time-lapse acquisitions by confocal fluorescence (CFM) and reflection (CRM) modes using confocal rheology (Fig. 2, D and E). We labeled an azide-functionalized PIC (LMW) with Alexa Fluor 488–DBCO (PIC-A488) (*44*) and monitored the formation of both networks during the temperature ramp (5 to 37°C). The fluorescence intensity (*I*^CFM^) emitted by PIC-A488 increased rapidly at lower temperatures showing a fast formation of the PIC network (movie S2). The fluorescence intensity (*I*^CFM^) remained constant, while the reflection intensity (*I*^CRM^) emitted by the collagen network increased only after being exposed to 37°C (Fig. 2D). This indicates that the formation of the PIC network precedes the assembly of the collagen network.

To demonstrate that there was no phase separation between the materials once they were formed, we performed coimage analysis and 3D reconstitution of the two networks (Fig. 2, F and G). We observed that the PIC was homogenously distributed and interpenetrated throughout the collagen network, forming an interpenetrating network (IPN) (movie S3). To further quantitatively evaluate the architecture of the collagen network in the presence of PIC, we characterized the structural properties of collagen in the composites by small-angle neutron scattering and ultrasmall-angle scattering (SANS–USANS) (Fig. 2H) and confocal laser scanning microscopy (CLSM) (fig. S3). Both methods demonstrate that the presence of PIC in the early stages of fibrillogenesis does not have an impact on collagen’s pore size or fiber diameter. In neutron scattering experiments, we first used contrast variation experiments to eliminate the scattering signal emitted by PIC. We found the contrast match point of PIC at 20% D_2_O and determined that collagen’s diameter (400 to 500 nm) (Fig. 2I) and pore size (1.5 μm) (Fig. 2J) remained consistent compared to pure collagen hydrogels. Analysis of the collagen’s pore size by CLSM also showed a similar trend, with collagen’s pore size (1.5 to 2 μm) remaining unaffected by increasing the PIC concentration (fig. S3). Together, these results demonstrate that the mechanical properties of collagen hydrogels can be tuned by using a low weight % (wt %) of PIC without affecting collagen’s architecture.

### PIC controls the stress-stiffening response of collagen networks

**Fig. 3.**
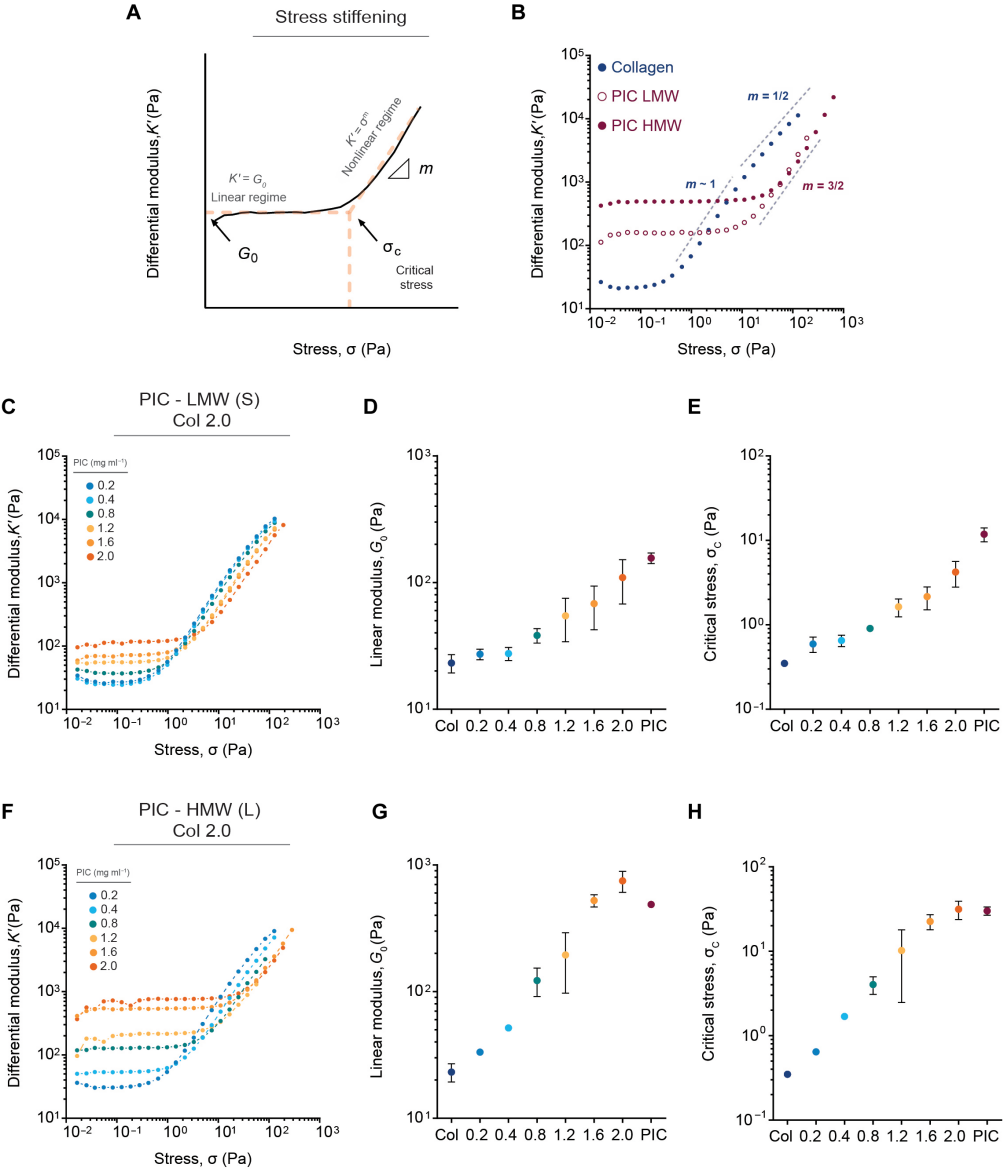
Nonlinear mechanical properties of PIC-Collagen IPNs. (**A**) Graphical representation illustrating a nonlinear elastic response characteristic of stress-stiffening materials. The response is described by the differential modulus *K′* (δσ*/*δγ) (Pa) as a function of applied stress (σ) (Pa). Stress-stiffening materials initially show a linear response at low levels of stress (*K′* = *G*_0_). The material stiffens following *K′* = σ^m^ (where m is the stiffening index) only when sufficient stress is applied to drive the material into the nonlinear regime. This transition is described by the critical stress (σ_c_). (**B**) Nonlinear response of pure bovine atelo collagen (2 mg ml^−1^, 1X PBS, pH 7) and PIC hydrogel (2 mg ml^−1^, 1X PBS) of LMW and HMW as a function of applied stress. (**C**) Differential modulus *K′* (Pa) of PIC-collagen composites containing a constant bovine atelo collagen concentration (2 mg ml^−1^) showing a stress-stiffening response. Increasing the density of the PIC (0.2 to 2.0 mg ml^−1^) using LMW PIC synergistically increases both (**D**) Linear elastic modulus (*G*_0_) and (**E**) critical stress (σ_c_) of bovine atelo collagen. (**F** to **H**) The synergistic mechanical effect is more pronounced when we increase the rigidity of PIC bundles with higher contour length (HMW PIC). Higher amounts of applied stress are required to deform a collagen network using larger PIC bundles [HMW (L)] compared to shorter PIC bundles [LMW (S)]. All datasets show an average of three independent measurements (*n* = 3).

After determining that the PIC does not influence collagen’s assembly or architecture, we next investigated how the semi flexibility of PIC can synergistically influence the response of atelo collagen networks to applied shear stress. To this end, we applied oscillatory shear stress σ (Pa) to the composites after gelation and evaluated changes in the nonlinear differential modulus (*K′*), particularly the onset of stiffening ( $\sigma_{c}$ ) (Fig. 3, A to H). When networks of reconstituted collagen stiffen with applied stress, they display a nonlinear response dominated by a bending (*m* ~ 1) to stretching transition (*m* ~ 1/ 2) (*6*–*8*). However, when combining collagen with LMW PIC, we first observed that, as the density of the LMW PIC increases, the onset of stiffening (σ_c_) of atelo collagen is shifted toward higher levels of stress (Fig. 3, C to E), meaning that atelo collagen becomes less responsive to stress. The shift in the nonlinear response becomes only apparent when reaching LMW PIC concentrations of 0.8 and 1.2 mg ml^−1^ (Fig. 3E). Coincidently, this range of PIC concentrations falls within the regime where PIC depicts a nonlinear response dominated by entropic fiber stretching (*m* ~ 3/2) (*37*). Moreover, our experiments using HMW PIC show that one can also decrease the sensitivity of collagen to applied stress by increasing the rigidity of PIC bundles (Fig. 3, F to H). The same decrease in sensitivity observed in composites with high concentrations of LMW PIC (2 mg ml^−1^) can be achieved with lower concentrations of HMW PIC (0.8 to 1.2 mg ml^−1^). Rescaling the stress-stiffening curves into a master curve indicates that all the composites are dominated by bending energy in the nonlinear regime (*m* ~ 1) (figs. S4 and S5) and that the entropic response (*m* ~ 3/2) of PIC is suppressed even when adding high concentrations of HMW PIC. Together, these results suggest that small additions of PIC control the stress-stiffening response of collagen networks by controlling the resistance of individual collagen fibers to bending.

### Investigating melanoma behavior in PIC-collagen composites

Next, we investigated biological responses of two different cell lines, melanoma, and fibroblasts, to changes in collagen’s nonlinear elasticity. Collagen-only hydrogels and other composite systems can tune collagen’s nonlinear behavior but often with changes in collagen concentration, architecture, or the introduction of multivalency. The combination of PIC with collagen offers an advantage over collagen-only hydrogels and other composite systems because the nonlinear mechanical behavior of collagen networks can be tuned independently. As PIC is also a biologically inert polymer, this composite system allowed us to investigate the response of cellular behaviors solely to changes in the stress-stiffening response of collagen networks.

**Fig. 4.**
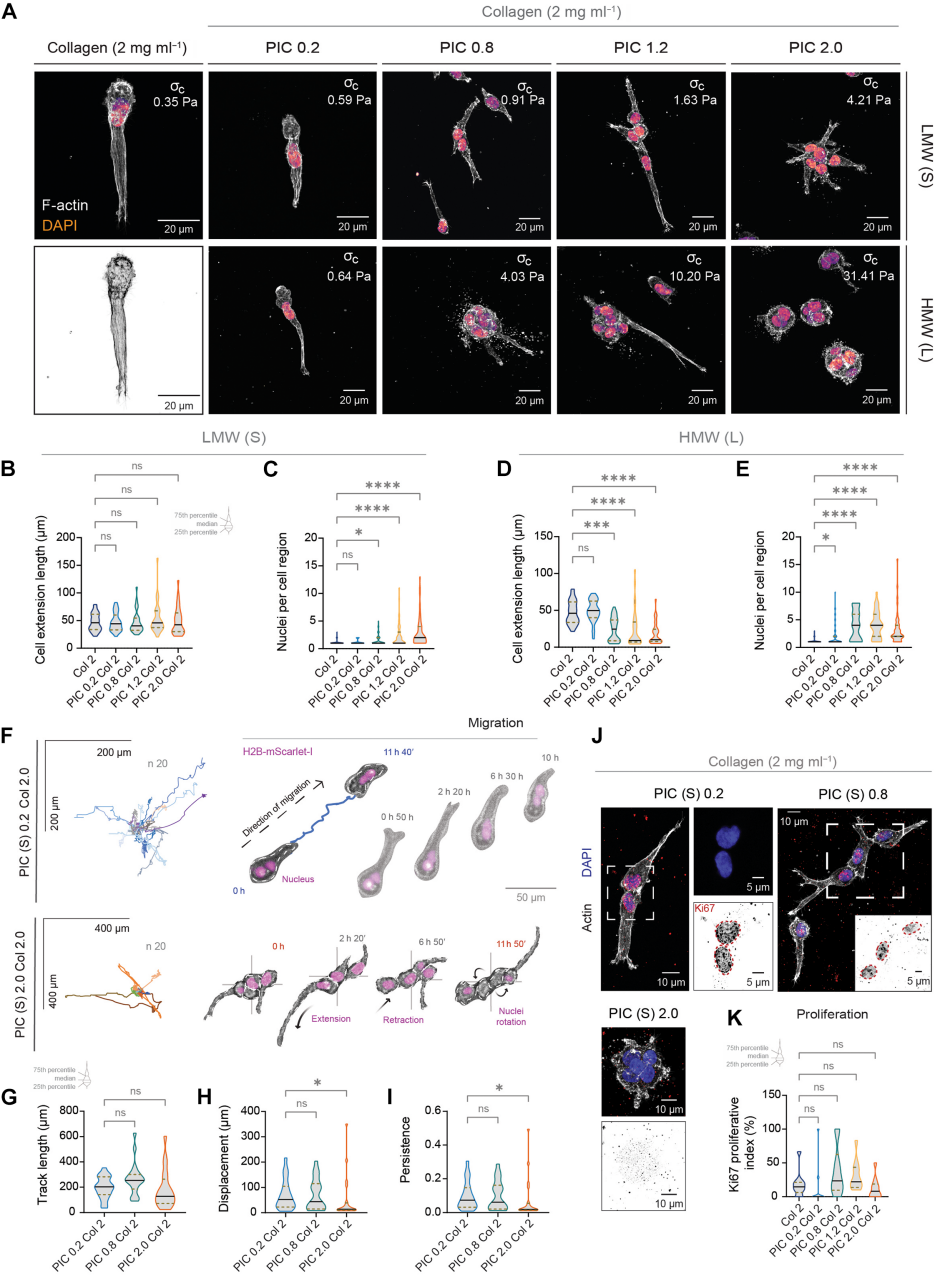
Tuning collagen’s rigidity with PIC modulates melanoma morphology and migration. (**A**) Confocal images of fixed metastatic melanoma (1205 Lu) cells in atelo collagen (2 mg ml^−1^) and LMW and HMW PIC-collagen composites (0.2 to 2.0 mg ml^−1^) after 6 days. (**B** to **E**) Quantification of [(B) and (D)] cell extension length (μm) and [(C) and (E)] number of nuclei per cell region in LMW and HMW PIC-collagen composites. Length is defined as the longest distance from the center of the nucleus to the tip of the pseudopodium. Number of nuclei quantified within a cell region. Value of one is an individual cell with a single pseudopodium. Values of two and above represent the number of nuclei within one cluster. Data obtained from three independent experiments (*n* = 30 to 72; *P < 0.05, ***P < 0.001, and ****P < 0.0001; ns, not significant). (**F**) Spiderplots of cell migration tracks in PIC-collagen composites (LMW PIC) with representative time-lapse images. Images acquired every 10 min for 12 hours. Cells were manually tracked (ImageJ) using both the center of the nuclei and perinuclear body in bright-field for reference. Time shown in hours (h) and min (′). Graphs display the 20 longest paths. (**G** to **I**) Migratory behavior quantified by (G) the track length (μm), (H) displacement (μm), and (I) persistence index ratio in each condition. Data were obtained from three independent biological experiments (*n* = 20; *P < 0.05). (**J**) Confocal images of fixed melanoma cells in PIC-collagen composites (LMW PIC) for 6 days and stained for Ki67. (**K**) Proliferative index (%) was obtained by quantifying the number of Ki67-positive cells per field of view over two independent experiments (*n* = 10). Scale bars, 10 μm and 5 μm (insets). All sets of data were analyzed by Kruskal-Wallis test with Dunn’s multiple comparison test.

We first investigated morphological responses of a highly motile metastatic melanoma cell line (1205 Lu) (Fig. 4). We embedded melanoma cells for 6 days in collagen-only and PIC-collagen composites before assessing their morphology (Fig. 4A; F-actin and nuclei). Consistent with previous observations (*45*), melanoma cells embedded in collagen-only hydrogels exhibited an elongated morphology with front-rear polarity, extending a singular actin-based pseudopod that allows cells to efficiently translocate within the collagen network (*46*, *47*). This morphology was conserved in composites with lower LMW PIC concentrations (0.2 mg ml^−1^), while concentrations of ~1 mg ml^−1^ and above induced cell clustering (Fig. 4A; 0.2 mg ml^−1^ versus 2 mg ml^−1^). While clusters exhibited an increase in the number of nuclei, cells were still able to form extensions of similar length in all concentrations of LMW PIC (Fig. 4, B and C).

When we embedded cells in collagen composites containing a higher PIC contour length (*L*_c_) (L, HMW) (Fig. 4, A, D and E), we also observed this morphological switch from single elongated cells to multicellular clusters. Our rheological experiments demonstrated that equivalent nonlinear mechanical properties of collagen hydrogels can be achieved by incorporating either higher concentrations of LMW PIC or lower concentrations of HMW PIC (Fig. 3). Hence, the clustering of cells in HMW PIC-collagen composites can also be induced with a significantly lower PIC concentration (Fig. 4A; LMW PIC of 2.0 mg ml^−1^, $\sigma_{c}$ of 4.21 Pa cf. HMW PIC of 0.8 mg ml^−1^, $\sigma_{c}$ of 4.03 Pa). Thus, the morphological switch is only triggered in cells embedded in composites exhibiting similar onsets of stiffening ( $\sigma_{c}$ ), indicating that the switch in morphologies may be induced when cells are required to exert similarly high levels of stress against collagen.

The morphological switch we observed further suggested that changes in collagen nonlinear mechanics may regulate the ability of cells to migrate. To examine the effect of our PIC-collagen composites on cell migration, we embedded single 1205 Lu melanoma cells and examined their morphology by time-lapse microscopy (Fig. 4, F to I ; 12 hours). While cells in all concentrations of LMW PIC were able to form cellular extensions and move within the composites (Fig. 4, F and G), cells in higher PIC concentrations (2 mg ml^−1^) failed to directionally migrate (Fig. 4, H and I). Instead, cells would cluster and rotate, producing short-lived cellular extensions, which failed to support the translocation of the cell body (Fig. 4F and movies S4 and S5). In contrast, cells embedded in lower concentrations of PIC were able to efficiently migrate (increased displacement and migration persistence) exhibiting longer-lived pseudopodia (Fig. 4F), demonstrating that the nonlinear mechanical behavior of collagen regulates pseudopodia stability and productive cell migration.

We hypothesized that changes in collagen mechanics may not only induce a migration switch but may also alter cell proliferation, accounting for the increase in the number of cell nuclei in composites with higher PIC concentration (Fig. 4, C and E). To test this, we cultured parental 1205 Lu cells in the PIC-collagen composites for 6 days, before immunostaining for the cell proliferation marker (Ki67) (Fig. 4, J and K). Examination of proliferation (Ki67-positive nuclei) revealed that cells were able to proliferate in all composites. However, clusters in high concentrations of PIC (2.0 mg ml^−1^) unexpectedly lacked Ki67, although they had the highest number of nuclei (Fig. 4J). Therefore, clusters are likely formed by an early proliferation event after embedding, which ceases once a critical cluster size is met. Together, these results show that increasing collagen’s onset of stiffening ( $\sigma_{c}$ ) controls the ability of cancer cells to migrate and proliferate.

### The onset of collagen’s stress-stiffening response ( $\sigma_{c}$ ) controls fibroblast morphology and collagen deformation

**Fig. 5.**
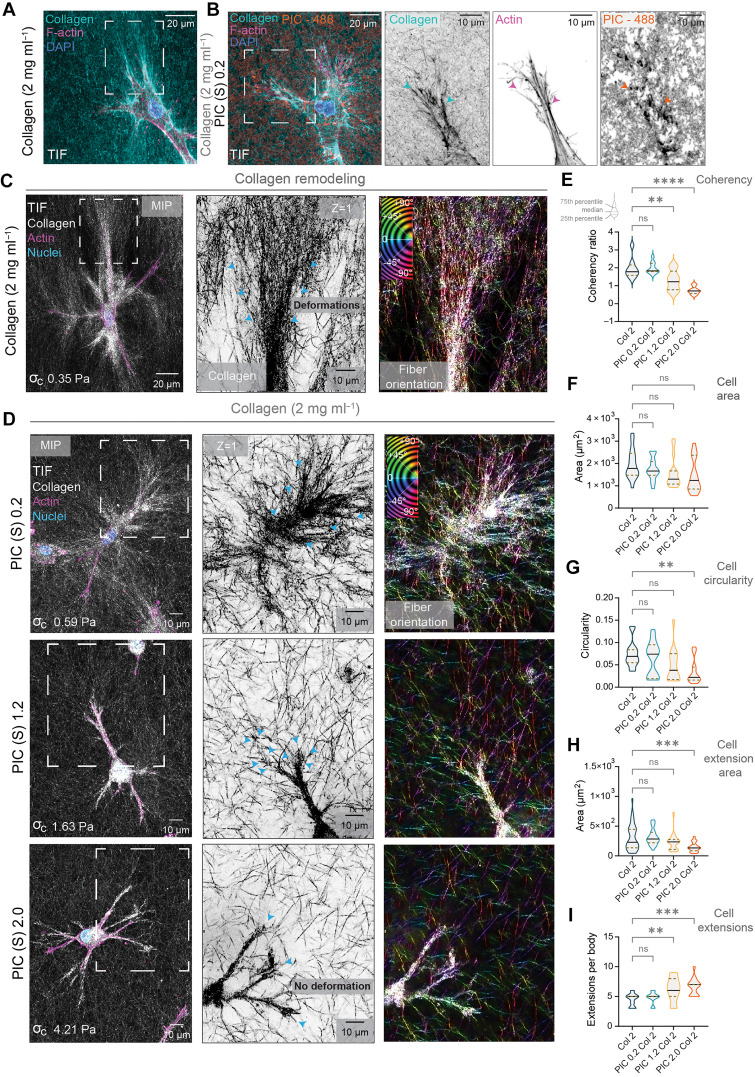
Fibroblast morphology correlates with collagen deformation and alignment in PIC-collagen composites. (**A** and **B**) Airy scan images of telomerase-immortalized fibroblasts (TIFs) in (A) atelo collagen (2 mg ml^−1^) and (B) a PIC-collagen composite (0.2 mg ml^−1^). Collagen (cyan) and PIC (orange) labeled with CNA35 mScarlet-I and PIC (S)–A488. *Z* MIPs show ~100 μm in depth. Images acquired at 37°C. (**C**) Confocal reflection images of a fibroblast in collagen (2 mg ml^−1^). MIP shows ~60 μm in depth. Inset shows a single Z slide (*Z* = 1) from a zoomed region of interest (dotted outline). Scale bars, 10 μm. Color-coded orientation (°, degrees) shows the orientation of the collagen fibers in the direction of the deformation. (**D**) Confocal reflection images of fibroblasts in PIC-collagen composites (S, LMW; 0.2, 1.2, and 2.0 mg ml^−1^; collagen, 2.0 mg ml^−1^). MIP are ~50 μm in depth. Inset shows a single Z slide (*Z* = 1) and color-coded orientation (°, degrees). (**E**) Quantification of collagen deformation with a coherency analysis. Data were obtained from two independent biological replicates (*n* = 9 to 10; ****P < 0.001; **P < 0.01; and ns, *P* > 0.05). Analysis first by a D’Agostino-Pearson omnibus normality test then with ordinary one-way analysis of variance (ANOVA) with Dunnett’s multiple comparisons. (**F** to **I**) Morphological quantification of fibroblasts in collagen-only hydrogels and PIC-collagen composites (S, LMW; 0.2, 1.2, and 2.0 mg ml^−1^; collagen, 2.0 mg ml^−1^). Quantification includes (F) total cell body area (μm^2^), (G) cell circularity, (H) cell extension area (μm^2^), and (I) the number of cell extensions per body. Data are obtained from two independent biological replicates (cell area, circularity, and cell extensions; *n* = 15; cell extension area, *n* = 40 extensions from 15 cells per condition; ***P < 0.001, **P < 0.01; ns, not significant). Analysis by Kruskal-Wallis test with Dunn’s multiple comparison test.

Next, to evaluate the effect of tuning collagen’s onset of stiffening ( $\sigma_{c}$ ) in the generation of cellular forces, we embedded contractile fibroblasts in our collagen composites (Fig. 5). During wound healing and tumor progression, fibroblasts exert contractile stresses via actomyosin-based contractility (*48*). These cellular deformations result in fibers being aligned in the direction of the cell’s leading edge (*14*). Thus, we first investigated changes in collagen remodeling by fibroblasts [telomerase-immortalized fibroblast (TIF)] with increasing LMW PIC concentration (0.2, 1.2 and 2.0 mg ml^−1^) (Fig. 5, A to D). We measured changes in collagen remodeling by analyzing the orientation of collagen fibers with a coherency analysis from confocal refection images (Fig. 5E) (*49*). We found that in composites containing lower concentrations of LMW PIC (0.2 mg ml^−1^), cells were able to actively deform and align collagen networks in a similar manner to cells embedded in collagen-only hydrogels (Fig. 5, A to E , and movie S6). However, when we increased the concentration of LMW PIC to 1.2 to 2.0 mg ml^−1^, the cell’s ability to deform and align collagen networks was reduced (Fig. 5, D and E). Collagen networks were more isotropic and less aligned in the direction of the cell’s leading edge. This was indicated by a decrease in collagen’s reflection intensity surrounding the tips of cellular extensions (Fig. 5D), a decrease in collagen coherency (Fig. 5E), and changes in cellular morphology (F-actin) (Fig. 5, F to I). While we did not observe a significant change in cell area (Fig. 5F), we found a decrease in cell circularity and in area of cellular extensions (Fig. 5, G to H). Cells became spindle-like in appearance with more thin cellular extensions (Fig. 5I). These changes in cell morphology along with the decrease in collagen remodeling indicate that small increases in $\sigma_{c}$ alter the cell’s ability to initially deform the collagen, limiting the transmission of force through the network over longer distances. Increasing the initial resistance to force in collagen networks may alter the formation of cell-matrix interactions.

### PIC-collagen IPNs tune cell-matrix interactions in 3D

Cells transmit mechanical forces from their surroundings via focal adhesions. Focal adhesions are multimolecular complexes of proteins that physically connect the rearward flow of intracellular F-actin with the extracellular environment via integrin-transmembrane receptors (*50*, *51*). It is recognized that the size and composition of focal adhesion complexes are influenced by the stimulation of tension generated by actomyosin contractility, acting as force-transmitting “molecular clutches” to facilitate forward cell movement (*52*, *53*). When cells move on glass, focal adhesions grow and elongate when they experience slower actin retrograde flow and tension in the lamella transition zone of the cell leading edge (*54*–*56*). However, despite this large body of research in two dimensions, less is known about the spatial organization of actin and focal adhesions when cells interact with 3D collagen networks.

**Fig. 6.**
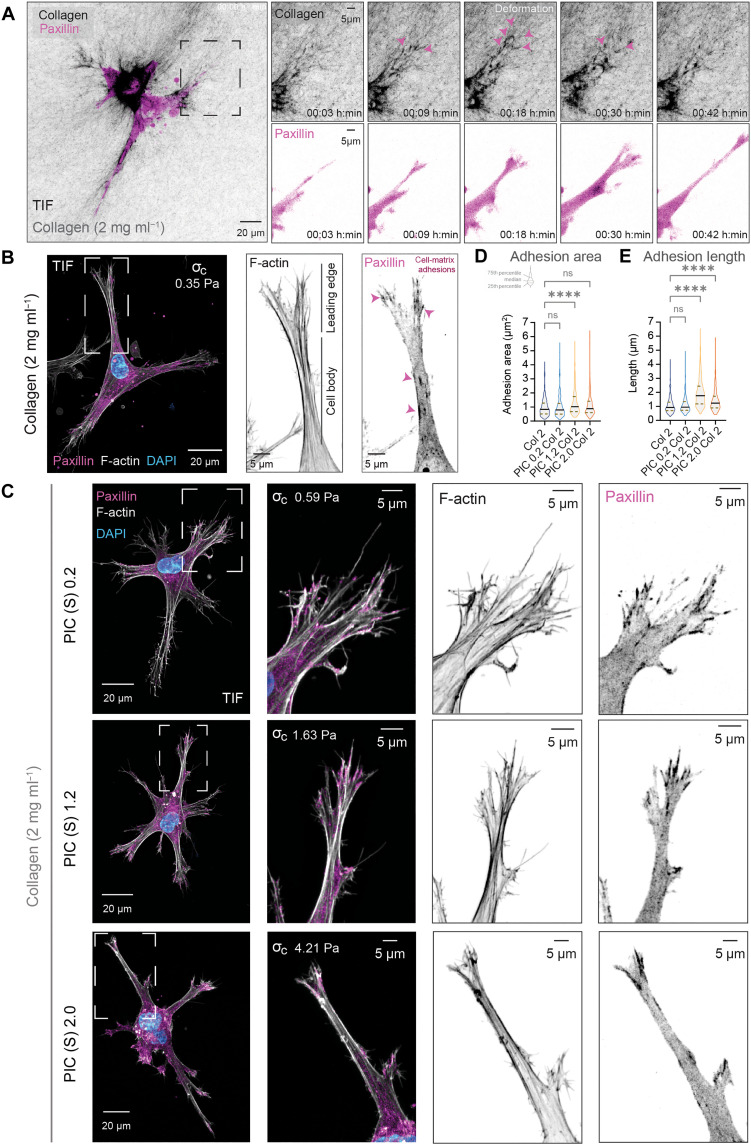
Tuning the onset of collagen’s stiffening ( $\sigma_{c}$ ) with PIC modifies cell-matrix adhesion morphology in 3D collagen hydrogels. (**A**) Time-lapse spinning-disc live-cell imaging of fibroblasts expressing paxillin-mCherry (magenta) showing remodeling of surrounding collagen (CNA35-eGFP) (gray) in collagen-only hydrogels (collagen, 2 mg ml^−1^; atelo bovine) after 24 hours. Scale bars, 20 μm and 5 μm (insets). Confocal images acquired with a 25× silicone objective (CFI Plan Apochromat Lambda S,1.05 N.A.) sequentially between stacks of 40 μm (2-μm step; 21 steps) for 3 hours every 3 min. (**B**) Confocal image of a fixed fibroblast-expressing paxillin-mCherry embedded in atelo collagen (2 mg ml^−1^) after 24 hours. MIP is ~50 μm in depth. Scale bars, 20 μm. Inset shows a zoomed region of a cell extension of interest. The extension is classified into two subregions: leading edge (LE) and cell body (CB). Scale bars, 5 μm. (**C**) Confocal images of fixed fibroblasts expressing paxillin-mCherry embedded in PIC-collagen composites (S, LMW; 0.2, 1.2, and 2.0 mg ml^−1^; collagen, 2.0 mg ml^−1^) after 24 hours. MIPs are ~50 to 100 μm in depth. Scale bars, 20 μm and 5 μm (insets). (**D** and **E**) Adhesion size increases at leading edge in response to changes in collagen mechanics. Adhesion size was quantified by (D) adhesion area (μm^2^) and (E) length (μm). Adhesions (*n* = 273 to 381) were manually quantified from two independent biological replicates (19 to 25 extensions from 10 to 14 cells per condition; ****P < 0.0001; ns, not significant). Analysis by Kruskal-Wallis test with Dunn’s multiple comparison test.

To investigate the effect of tuning the onset of collagen stiffening ( $\sigma_{c}$ ) on the spatial organization of actin and focal adhesions, we compared fibroblasts stably expressing the focal adhesion marker Paxillin-mCherry, embedded in collagen-only and composite hydrogels (Fig. 6). We chose the adapter focal adhesion protein paxillin because it is present throughout the focal adhesion turnover cycle, at both nascent and maturation stages (*57*–*59*), ensuring we would capture adhesions. In collagen-only hydrogels, fibroblasts deform collagen networks when they form cellular extensions, consisting of (i) a peripheral leading edge, which is the tip region, rich in fine actin extensions known as filopodia and (ii) a cell body (CB), which is the pseudopod neck-like region adjacent to the leading edge in front of the nucleus (Fig. 6, A and B; fig. S6; and movie S7). We observed a higher accumulation of actin at lateral regions of the leading edge compared to the central region (fig. S6). Consistent with previous reports (*60*), we also observed that paxillin localizes in small puncta at the ends of actin bundles with adhesions that are aligned in the orientation of these actin structures (fig. S6). However, adhesions localized in the neck or lateral region of cell extensions were elongated (Fig. 6B and fig. S6). This suggests that when cells are embedded in collagen-only hydrogels, cell-matrix interactions at the leading edge are weak, resulting in small punctate adhesions that likely undergo rapid turnover.

In comparison, in the PIC-collagen composites, we found that paxillin exhibited a biphasic morphology with a change in spatial distribution in response to decreasing the sensitivity of collagen to applied stress (Fig. 6, C to E). In collagen composites containing LMW PIC (0.2 mg ml^−1^), adhesions were small, similarly to those of cells embedded in collagen-only hydrogels (Fig. 6, C to E). However, when we increased the concentration of LMW PIC to 1.2 mg ml^−1^, the size of the adhesions at the tips of the leading edge increased significantly compared to the cell body (Fig. 6, C to E, and fig. S7), indicating that cellular tensional forces increase at the leading edge with small increases in collagen’s $\sigma_{c}$ . However, further increasing collagen’s $\sigma_{c}$ with higher concentrations of LMW PIC (2.0 mg ml^−1^) resulted in a decrease in adhesion size at the tips of the leading edge (Fig. 6, D and E). This suggests that adhesions grow and elongate at the leading edge only for a limited increase in $\sigma_{c}$.

To further evaluate the relationship between cell adhesion forces with changes in collagen’s $\sigma_{c}$ , we analyzed the displacement of fluorescent microbeads surrounding fibroblasts by live-cell imaging (fig. S8 and movie S8). Consistent with our previous observations (Fig. 5), cells were able to form extensions in all composites, but the ability to deform collagen networks decreased with increasing LMW PIC concentration. Beads in composites with 2 mg ml^−1^ PIC had the smallest displacements. However, we found that, while the bead displacements were similar in composites containing 0.2 and 1.2 mg ml^−1^ PIC, the rate of the displacement decreased in composites above 1.2 mg ml^−1^ PIC (fig. S8, B and C), suggesting a more prolonged application of force. The altered ability of the cells to displace the beads and the changes in adhesion morphology indicate that the initial interaction between cells and the collagen network strengthens with small increases in $\sigma_{c}$ . However, it subsequently weakens after a force-limited point. This is likely due to molecular clutches physically disengaging by frictional slippage, consistent with recent postulated rate-dependent mechanisms of force loading (*61*).

Our observations suggest that the onset of collagen stiffening ( $\sigma_{c}$ ) is likely key in modulating the initial force loading rate within focal adhesions during initial cellular contractions. We hypothesize that the small increases in collagen’s $\sigma_{c}$ allow the network to be rigid enough for individual clutches at the cell’s leading edge to interact with the network at a rate that enables an effective binding (on rate). However, increasing collagen’s $\sigma_{c}$ above an optimal point prevents cells from applying stress efficiently and driving the network into a nonlinear stress-stiffened regime. The decrease in adhesion size and bead displacement in composites with higher $\sigma_{c}$ indicate that the size of this initial force results in forces being loaded too rapidly within individual clutches. This stimulates faster unbinding rates (off rate) and may explain the reduced ability of cells to contract rigidified collagen networks.

## DISCUSSION

Our work presents a tuneable composite system where the mechanical properties of collagen hydrogels can be easily manipulated with the synthetic polymer, PIC. We have shown that, by only introducing small concentrations and different contour lengths (*L*_c_) of PIC, we can influence the mechanical behavior of collagen hydrogels without changing the reconstitution process of collagen. This system can rapidly undergo gelation by forming a semi flexible background of extremely low synthetic polymer density. This low polymer density mechanically supports the polymerization of collagen networks, forming an IPN. The sequential assembly of this IPN system is a process that is entirely driven by physiological heat (37°C), making it an optimal system for 3D cell culture where cells can be rapidly encapsulated in 3D collagen hydrogels.

By combining collagen with PIC, we also demonstrate that cells respond to small changes in collagen’s nonlinear elasticity. The unique ability of fibrous materials to stiffen as they are strained has long been regarded as an important mechanical regulator of cell-matrix interactions and cell force generation (*15*, *16*, *62*). However, in contrast to previous studies where the architecture or concentration of collagen is commonly changed, the introduction of small PIC concentrations allowed us to solely investigate the role of collagen’s onset of stiffening ( $\sigma_{c}$ ) on biological behaviors. The introduction of very small polymer weight density (0.2 to 2.0 mg ml^−1^ being 0.02 to 0.2 wt %) of PIC into collagen hydrogels allows us to tune collagen’s nonlinear elasticity while maintaining similar linear mechanical profiles (*G′* and viscoelasticity) and a constant collagen architecture.

Previous studies have shown that the amount of stress applied solely by cellular deformations is not large enough to stress-stiffen fibrous materials to the point of breaking or rupturing the material. Rather, fibrous materials stress-stiffen beyond the cell’s ability to contract, and cells can only stiffen fibrous materials during the early stages of the stress-stiffening regime (*15*, *63*). Our results align with these early observations, demonstrating that cells acutely respond to alterations in $\sigma_{c}$ (the initial amount of force required to initiate a resistance against collagen). Increasing $\sigma_{c}$ decreases the ability of the cells to apply resistance, in turn regulating biological behaviors in 3D environments such as migration, proliferation, and the cell’s ability to contract collagen networks over long distances.

In the context of cell motility, we found that when collagen is rigidified by synergistic mechanical interactions with a second polymer network, migration becomes nonproductive with a morphological switch. We observed that small increases in collagen’s $\sigma_{c}$ disrupts pseudopodia formation, decreasing persistent migration and resulting in cells forming small clusters. We found that this morphological switch to clustering is dependent on the rigidity of collagen when combined with extremely low concentrations of stiffer PIC polymers. This observation suggests that the nonlinear elasticity of collagen dictates cell migration independently of collagen network density or architecture.

In mesenchymal migration, the formation of the pseudopodium in 3D collagen is dependent on focal adhesion complexes formed by integrin-based interactions (*64*). As these complexes physically link the cellular cytoskeleton to the extracellular environment and mature as a function of force, we hypothesized that increasing the rigidity of collagen networks influenced cell-matrix interactions. To this end, we investigated the effect of tuning collagen’s nonlinearity on focal adhesions in fibroblasts. Previous studies have shown that integrin-based adhesions cluster when tuning the rigidity of collagen with different collagen architectures (*65*). However, our results demonstrate that the ability of fibroblasts to actively contract collagen networks is directly influenced by a biphasic response of focal adhesions to small changes in $\sigma_{c}$ . Consistent with recent postulated mechanisms of force loading across components of the molecular clutch (*61*, *66*), we observed that adhesions elongate in collagen hydrogels only at the point at which collagen networks are rigid enough, but still malleable, for cells to actively contract. We entered this mechanical regime, or optimal rigidity, experimentally when combining collagen with LMW PIC (~1 mg ml^−1^). By investigating the spatial organisation of actin and paxillin at the leading edge of cells, we observe that adhesions elongate at the tips where actin bundles accumulate. Increasing the rigidity of collagen with PIC above this mechanical regime results in a decrease in adhesion size, suggesting rapid unbinding events and bond destabilization within focal adhesions.

We speculate that this biphasic response of focal adhesions may also result in different rates of actin flow with increasing tension at the leading edge, modulating the rate of focal adhesion turnover. Thus, it will be critical for future investigations to determine the spatial localisation of mechanosensitive force transduction proteins such as vinculin and talin. Key to this will be gentle, advanced volumetric imaging techniques capable of achieving subcellular resolution of focal adhesion turnover dynamics in 3D (*67*). In addition, future experiments using traction force microscopy will determine not only specific amounts of force exerted against collagen with variations in $\sigma_{c}$ but also whether there are differences in the rate at which force is being applied. Ultimately, these will couple our understanding of the kinetic behavior (on/off rates) of focal adhesion complexes when cells initially generate contractile force to stiffen networks of collagen.

Further field application of this 3D composite system will facilitate the exploration of morphological responses that are not commonly observed in collagen-only hydrogels and the molecular mechanisms that underpin them. In the context of tissues, our findings provide a deeper insight into how cells respond to an increase in tension as they encounter larger depositions of matrix during wound healing and pathological conditions such as cancer.

## MATERIALS AND METHODS

### Synthesis of PIC (LMW, HMW, PIC-N_3_, and PIC-Alexa Fluor 488)

Following a previously described procedure for the synthesis of PIC (*36*–*38*), polymers containing methoxy-functionalized triethylene glycol (EG_3_) monomers were synthesized using 1:1000 (LMW; S, short polymer) and 1:10,000 (HMW; L, long polymer) catalyst/monomer ratios. Polymers were characterized by viscometry (LMW *M*_v_: 283 kg mol^−1^, HMW *M*_v_: 518 kg mol^−1^) and rheology in 1X PBS. LMW PIC-azide polymers were synthesized as described using an EG_3_-azide functional monomer in a 1:100 azide-to-methoxy ratio (*M*_v_: 170 kg/mol). LMW PIC-azide was reacted with AlexaDye 488–DBCO as previously reported with minor modifications (*44*). LMW PIC-azide (100 mg) was dissolved in 50 ml of acetonitrile (ACN) for 48 hours at room temperature. Then, a solution of AlexaDye 488 DBCO (3.123 × 10^−7^ mol, 0.3108-mg dye) from a stock solution (2.99 mM *N*,*N*′-dimethylformamide) was added to the PIC solution and stirred for 48 hours at room temperature while avoiding light exposure. The polymer was precipitated in cold diisopropyl ether, purified by dialysis [molecular weight cut-off (MWCO) of 14 kDa] at 4°C for 24 hours against MQ water and recovered by lyophilization.

### Preparation of collagen solutions

All reagents were cooled on ice before use. Collagen type I atelo of bovine origin [nutragen; (5.9 mg ml^−1^ in 0.01 M HCI); #5010, Advanced Biomatrix] was diluted with one-part 10X PBS, followed by neutralization to pH 7 with 0.1 M NaOH and diluted with MQ water to generate solutions of 4 mg ml^−1^.

### General preparation of PIC-collagen composites

All experiments were performed using the methoxy-functionalized EG_3_ PIC polymers unless stated otherwise. To form the PIC-collagen composites, PIC of LMW or HMW was dissolved in 1X PBS to generate a stock solution of 4 mg ml^−1^. The PIC solutions were dissolved in an automatic rotator at 20 rpm for 72 hours at 4°C. The stock solution of PIC was diluted in a series of dilutions with cold 1X PBS to produce solutions of 200 μl of final volume. Next, 200 μl of the prepared PIC solution (0.4, 0.8, 2.4, 3.2, and 4.0 mg ml^−1^) was added to 200 μl of a cold solution of collagen (4 mg ml^−1^, 1X PBS) to give composites with final PIC concentrations of 0.2, 0.4, 0.8, 1.2, 1.6, and 2.0 mg ml^−1^ while containing a final collagen concentration of 2.0 mg ml^−1^. The composites were mixed by pipetting while swirling to avoid bubbles and kept on ice before their characterization.

### Rheology

Collagen gels (2 mg ml^−1^) and PIC (0.2 to 2.0 mg ml^−1^)–collagen (2 mg ml^−1^) composites were analyzed using an Anton Paar MCR-502 WESP rheometer. Samples were transferred to a precooled (5°C) bottom stainless steel plate (Anton Paar) setting a gap of 0.5 mm using a stainless steel parallel plate PP-25 (25-mm diameter; Anton Paar). Samples were trimmed, and a thin layer of silica oil was placed around the sample. Gelation was induced with a temperature ramp from 5° to 37°C (1°C min^−1^) and further incubated for 2 hours at 37°C. Changes in viscoelastic properties were monitored by applying oscillatory shear rheology at a constant strain of 0.5% and a frequency of 1 Hz. A frequency sweep from 10 to 0.1 Hz at constant strain of 0.5% was then applied and the nonlinear profile was evaluated using previous prestress protocol (*36*). The linear elastic modulus *G*_0_ represents an average of the first three individual values in the linear regime at low levels of stress. Following previous studies (*37*, *38*), we obtained the onset of stiffening (σ_0_) by performing a linear interpolation of the differential modulus *K′* (δσ*/*δγ) curve for each condition and determined the minimal stress values.

### Confocal rheology

Simultaneous confocal imaging and rheology was performed with a custom built confocal rheometer by manually sliding a rheometer with an open bottom plate configuration (MCR-502 WESP rheometer, Anton Paar) placed on a rail stage until positioned on top of an inverted confocal microscope (TCS SP8 WLL, Leica Microsystems) (*68*, *69*). All measurements were performed with oscillatory shear rheology and a measuring stainless steel parallel plate PP-25 (25 mm; Anton Paar). An extension tube was used for installing the objectives under the rheometer stage. For assembling the rheometer sample holder, in general, a 3-mm plastic ring was placed under a thin steel plate with an objective hole. A glass cover slip (50 mm, #1.5) was placed on top of the thin steel plate and sealed with sealant ring (rubber) and stainless streel ring with rubber ring.

#### Confocal radial distance calibration

The radial distance of the objective was calibrated within ~1 mm form the center of the plate to reduce visualized motion from the oscillation. To calibrate, scans of 512 pixels by 512 pixels (465 μm by 465 μm) were acquired with a 20× Air objective using reflection mode and gap between plates of 0.1 mm. A 488-nm laser with an emission range of 479 to 498 nm using a photomultiplier tube (PMT) detector was used to acquire the reflection from the bottom surface of the plate. A steady shear rate of 0.3330 (s^−1^) was applied while simultaneously imaging at a rate of 27.87 (s^−1^) for 30 s. Distinguishable defects on the surface of the plate were used to calculate the radial position of the objective by calculating the local linear velocity.

#### Image acquisition

After calibration, a HC PL APO CS2 40×/1.10 water immersion objective (Leica Microsystems) with correction collar of 0.18 was placed under the lower plate, and glass cover slips were precooled to 5°C with a gap of 0.5 mm for 10 min before placing each sample. After placing the samples, the measuring gap was set to 0.5 mm, and the samples were trimmed, and a thin layer of silica oil was placed around to avoid dehydration. Fields of views were set to at least ~50 μm above the bottom glass. The reflection intensity of the collagen fibers was acquired using a 550-nm laser and an emission range of 530 to 570 nm with a PMT detector. Stacks of 20 μm in size were acquired with a step size of 1.0 μm every 1 min for 2.5 hours. All scans were 1024 pixels by 1024 pixels (61.44 μm by 61.44 μm) and acquired in a bidirectional scan direction with a line accumulation of 2. Time zero is defined as the time when the starting temperature is 5°C. The general protocol for inducing gelation with temperature ramp (5° to 37°C, 1°C min^−1^) and incubation for 2 hours at 37°C was applied, and changes of viscoelastic properties (*G′* and *G″*) were monitored over time.

#### Co-imaging of fluorescence and reflection modes

To acquire both channels at the same rate as the temperature increase (1°C min^−1^), stacks of 10 μm in size were acquired with a step size of 1.0 μm every 1 min for 2.5 hours. The fluorescence intensity of the PIC-A488 was acquired using a 488-nm laser and an emission range of 510 to 560 nm. The reflection intensity of the collagen fibers was acquired using a 650-nm laser and an emission range of 630 to 670 nm with a PMT detector. Scans were acquired sequentially between stacks while collecting the reflection of the fibers in the first line. All scans were 1024 × 1024 pixels (61.44 μm by 61.44 μm) and acquired in a bidirectional scan direction with a line accumulation of 2. Time zero is defined as the time when the starting temperature is 5°C. After gelation for 2 hours, images of 20 μm in depth from three different regions were acquired at 37°C using this setup to maintain the formation of the PIC network. Maximal projections were processed in Fiji, and 3D reconstructions were processed in Imaris ×64 (version 9.7.2).

### Pore analysis by confocal laser scanning microscopy

Collagen and PIC–collagen composites (1X PBS) were imaged by confocal reflection microscopy using an inverted confocal microscope (TCS SP8 WLL, Leica Microsystems). Hydrogels were incubated at 37°C for 2 hours in a 15-well chamber slide (ibidi, USA) and heating block. Samples were imaged at room temperature (~21°C) after gelation. A 488-nm laser and an emission range of 478 to 498 nm using a HC PL APO CS2 40×/1.10 water immersion objective (Leica Microsystems) with correction collar of 0.18 and hybrid detector (HyD) were used to acquire data. To evaluate homogeneity throughout the samples, scans were acquired ~50 μm from the bottom layer, and three consecutive stacks of 50 μm size were acquired with a step size of 1.0 μm. All scans were 1024 by 1024 pixels (235 μm by 235 μm). Maximal projections were generated in Fiji by applying a mean and Gaussian blur filter filtering out features smaller than 1 pixel before binarization. An automated analysis was used to determine the pore size of the collagen networks using open-source MATLAB algorithm (*70*, *71*).

### Small-angle neutron scattering–ultrasmall-angle neutron scattering

Combined SANS and USANS techniques were used to determine the structural properties of the collagen networks with a linear increase in PIC concentration. Both SANS (QUOKKA) (*72*, *73*) and USANS (KOOKABURRA) (*74*) were performed at the Australian Centre for Neutron Scattering at the Australian Nuclear Science and Technology Organisation (Sydney, Australia).

#### Sample preparation

All neutron scattering experiments were performed using the methoxy-functionalized EG_3_ PIC polymers of LMW (*M*_v_: 283 kg mol^−1^) in 1X PBS. Collagen solutions using bovine atelo type I collagen [nutragen (5.9 mg ml^−1^); Advanced Biomatrix] were prepared at 4 mg ml^−1^ in 1X PBS. For SANS and USANS experiments, 500 μl and 2.0 ml of sample were prepared, respectively.

#### SANS-USANS acquisition

All SANS and USANS measurements were performed at 37°C. For SANS, a *q* range of 7 × 10^−4^ to 0.1 Å^−1^ was covered using an incident wavelength of 6 Å with 10% resolution. Three instrument configurations were conducted at source-to-sample (SSD) = sample-to-detector distances (SDD) = 20 m, SSD = SDD = 8 m, and SSD = 4 m, while SDD = 1.3 m. The source and sample aperture diameters were 50 and 12.5 mm, respectively. Samples, loaded in quartz cuvettes with 2-mm thickness, were measured. For USANS, samples were measured using a 29-mm aperture and neutron wavelength of 4.74 Å, covering a *q* range of 4 × 10^−5^ to 8 × 10^−4^ Å^−1^. SANS data were then reduced and merged with desmeared USANS data as previously described (*73*, *75*, *76*), and the combined data were plotted. Data fitting and analysis were conducted on SASView 5.0.5. For the structural analysis of fiber diameter, the scattering data probed in the high *q* range (SANS) were analyzed using a modified Guinier model following Eq. 1, where *I*(*Q*) is the scattering intensity at scattering vector (*Q*) = (4π/λ) sinθ, and 2θ is the scattering angle and *R*_g_ is the radius of gyration

$$I\left(Q\right)=\frac{I\left(0\right)}{Q}\text{exp}\left(-\frac{Q^{2}{R_{g}}^{2}}{2}\right)\;$$ (1)

Similarly, the pore sizes of the networks were analyzed by the same model using the scattering data in the USANS range. Average fiber diameter (*D*) or average pore size ( ξ ) was calculated from *R*_g_ using Eq. 2

$$D\;\text{or}\;\xi =2\times R_{g}\times \sqrt{2}\;$$ (2)

#### Contrast variation

To separate the scattering intensity of PIC from the collagen in the PIC-collagen composites, we first performed a neutron contrast variation experiment using SANS (QUOKKA) and determine the match point of the PIC. To this end, we measured the neutron scattering of a series of solutions where we linearly increase the concentration of deuterium oxide (D_2_O) in PIC solutions of 2 mg ml^−1^. We prepared the solutions with different ratios of D_2_O/H_2_O (0, 20, 40, 50, 60, 80, and 100% D_2_O) using ratios of two 100.0-ml solutions of 1X PBS (one solution using 100% MQ water and another using 100% D_2_O). We observed that the match point of PIC was at 20% D_2_O. Once we determined the match point, we prepared stocks of PIC (2 and 4 mg ml^−1^) by dissolving the polymers in a solution of 40% D_2_O containing 1X PBS in an automatic rotator at 20 rpm for 72 hours at 4°C. On site, we diluted the stock solutions using a solution containing 40% D_2_O/1X PBS to generate PIC solutions of 0.4, 2.4, and 4.0 mg ml^−1^ (1X PBS) and combined equal amounts of volume to a solution of collagen prepared with 100% MQ using 1X PBS as previously prepared for the other experiments to generate a final 20% D_2_O solvent. Note that for the contrast variation experiments requiring higher percentages of D_2_O, these were prepared from a stock solution of PIC in 100% D_2_O/1X PBS and from dialyzing the collagen stock solution in 100% D_2_O. For the dialysis, we transferred ~5 ml of collagen solution to a dialysis tubing membrane (SnakeSkin; MWCO of 7000, 22 mm–by–35 feet dry diameter) and dialyzed against 50 ml of 100% D_2_O with 0.02 M acetic acid in a falcon tube overnight at 4°C. We observed no precipitation when recovering the collagen from the membrane. To reconstitute the collagen in 90% D_2_O, we added 1 part of 10X PBS (MQ water) after mixing the collagen stock first with the PIC solution (100% D_2_O, 1X PBS), 0.1 M NaOH (MQ water), and the corresponding amount of D_2_O to make up the final volume.

### General cell culture

Human melanoma cell lines (1205 Lu) and telomerase-immortalized fibroblasts (TIFs) were cultured in T75 cell culture flasks with 10.0 ml of a general cell culture media solution of 500 ml Dulbecco’s modified Eagle’s medium (DMEM; high glucose, pyruvate, no glutamine; Gibco, 10313021) containing 50 ml of 10% of fetal bovine serum (Gibco, 10100147), 5 ml penicillin-streptomycin (10,000 U/ml; Gibco), and 5 ml Minimum essential medium non-essential amino acids (1000×, Gibco). The 1205 Lu endogenously labeled for monomeric enhanced green fluorescent protein (meGFP)–α-tubulin (CRISP)–expressing mScarlet-I H2B (*45*) and TIFs expressing Paxillin-mCherry was established by lentiviral transduction (*59*, *77*). TIF cells were cultured in blasticidin S hydrochloride (10 μg/ml; Gibco, A1113903).

### General procedure for cell embedding in PIC-collagen composites

All collagen and PIC solutions were prepared following the sample preparation used for rheology experiments using sterile solutions of PBS (10X, 1X) and MQ water. Note that for cell culture experiments, we used a 12-μl sterile solution of 7.5% sodium bicarbonate (Gibco) to neutralize the collagen solution to pH 7. We adjusted the volume with 32.4 μl of sterile water to make a final volume of 200 μl. The amount of 10X PBS was kept consistent. All reagents were kept in ice before embedding. In general, cells were detached and resuspended in 1 ml of media. A cell pellet of 5.0 × 10^4^ cell ml^−1^ was generated by quickly centrifuging the solution containing the cells in 1.5-ml Eppendorf tube at high speed for 10 s. Medium was removed slowly without disturbing the cell pellet. The cold collagen solution (4 mg ml^−1^, 1X PBS, pH 7) was added on top of the pellet and mixed by pipetting while swirling. The solution was placed momentarily in ice to prevent polymerization. The collagen solution (40 μl) containing the cells were mixed by pipetting with 40 μl of the desired PIC solution in a separate 1.5-ml Eppendorf tube. The material/cell mixture (30 μl) was transferred to a well from a preheated (37°C) μ-Slide 18 Well Glass Bottom dish (# 81817, Ibidi). The samples were incubated for at least 2 hours in a humidified chamber at 37°C/ 5% CO_2_. General cell culture DMEM (70 μl) containing 20 mM Hepes was added on top of hydrogels and incubated at 37 C/5% CO_2_ before imaging or fixation.

### General procedure for fixation

All cells were fixed by removing the cell media and adding 70 μl a solution of 4% paraformaldehyde (PFA) in 1X Brinkley Buffer 1980 [5X BRB80; 400 mM pipes, 5 mM MgCl_2_, and 5 mM EGTA (pH 6.8)] and MQ water for 15 min at room temperature. The fixation solution was removed, and the samples were washed twice with 70 μl of 1X PBS^+^ (0.9 mM Ca^2+^ and 0.5 mM Mg^2+^) for 5 min. The 1X PBS (70 μl) was added to the samples before staining or for storage at 4°C.

### 1205 Lu in PIC-collagen composites

#### Live-cell imaging experiments

Cells were imaged by live-cell imaging 72 hours post- encapsulation. Imaging was performed in an Andor Dragonfly Spinning Disc Scanhead confocal microscope with dual Andor Zyla 4.2 scientific complementary metal-oxide semiconductor (sCMOS) cameras equipped with CO_2_ and temperature-controlled chamber (37°C) and a light-emitting diode (LED) Cool/LED pe-100 illumination system. Time-lapse imaging was acquired with a 20× dry objective [Plan Apo VC, 0.75 numerical aperture (N.A.)] by sequentially acquiring fluorescent channels 488 mm (30%, BP 525/50; 200 ms, confocal pinhole of 25 μm) and 561 mm, (20%, BP 600/50; 200 ms, confocal pinhole of 25 μm) and bright-field channels (15% intensity LED Cool/LED pE-100; 400 ms, confocal pinhole of 25 μm). Scans were acquired every 10 min for 12 hours using a fully monotonized X-Y-Z stage capturing scans in at least two different regions per sample. Cell tracking analysis was performed using the Manual Tracking plugin in Fiji to determine the track length (μm), displacement (μm), and persistence traveled in each condition.

#### Proliferation experiments

1205 Lu parentals were embedded in collagen and PIC-collagen composites for 6 days and fixed. After fixation, cells were permeabilized with 70 μl of 0.5% Triton X-100/PBS for 10 min at 4°C and blocked in 70 μl of a solution containing PBS^+^, saponin, and fish skin gelatin (PFS) (500 ml of PBS^+^, 3.5 g of fish skin gelatin, 1.25 ml of 10% saponin/PBS, and 0.02% NaN_3_) for 1 hour at room temperature while gently rocking. The primary antibody [rabbit polyclonal to Ki67 (1:250); ab15580, Abcam) was diluted in PFS. The antibody solution (50 μl) was added to each well and incubated for 2 hours at room temperature while gentle rocking. Samples were washed three times by incubating 70 μl of PFS for 5 min at room temperature while gently rocking. Next, the secondary antibody Janelia Fluor 549 [polyclonal anti-rabbit IgG (H+L), 1:200; NBP1-75286JF549, Novus Biologicals] and Alexa Fluor 488 phalloidin (1:500; A12379, Invitrogen) were diluted in PFS. The solution (70 μl) was added to each well and incubated for 2 hours at room temperature while gently rocking and avoiding light exposure. Samples were washed three times by incubating 70 μl of PFS for 5 min at room temperature while gently rocking. A 4′,6-diamidino-2-phenylindole (DAPI) solution in 1X PBS (70 μl; 1:10,000; D1306, Invitrogen) were added to each well and incubated for 10 min at room temperature while gently rocking and avoiding light exposure. Samples were washed with 70 μl of 1X PBS for 5 min at room temperature while gently rocking and stored with 70 μl of 1X PBS at 4°C. Imaging was performed in an Andor Dragonfly Spinning Disc Scanhead microscope with a 40× water objective (Apo λ LS, 1.15 N.A.) using GenTeal gel (Alcon Laboratories) for immersion to avoid rapid evaporation of water. Images were acquired sequentially in the following order: 561 nm (50%, BP 600/50; 400 ms, confocal pinhole of 25 μm), 488 nm (10%, BP 525/50; 200 ms, confocal pinhole of 25 μm), and 405 nm (20%, BP 450/50; 200 ms, confocal of 25 μm). Stacks of 50 to 70 μm in size were acquired with a step size of 0.2 μm while acquiring all *Z* positions for each channel. At least five different regions were acquired for each condition. All scans were 2048 pixels by 2048 pixels (16-bit), binning 1 by 1 with a frame averaging of 1.

#### Image processing

After acquisition, images were maximally projected in Fiji and deconvolved using Microvolution Deconvolution (version 2016.02). In general, 10 deconvolution iterations were run per channel, and the background was corrected at 1%. A confocal PSF model was specified. The vectorial model was used as the mathematical approximation of microscope diffraction. A pinhole spacing of 625 nm and back-projected pinhole radius of 312.5 nm were specified for all emission wavelengths.

### Human dermal fibroblasts in PIC-collagen composites

#### Cell-matrix adhesion experiments

TIF cells expressing Paxillin-mCherry were embedded in collagen and PIC-collagen composites for 24 hours before fixation. Alexa Fluor 488 phalloidin (1:500; A12379, Invitrogen) and DAPI (1:10,000; D1306, Invitrogen) were diluted in 1X PBS directly added to the sample without permeabilization. Samples were incubated for 2 hours at room temperature while gently rocking before washing three times with 1X PBS as previously reported. Imaging was performed in an Andor Dragonfly Spinning Disc Scanhead microscope with a 40× water objective (Apo λ LS, 1.15 N.A.) using GenTeal gel (Alcon Laboratories). Images were acquired sequentially in the following order: 561 nm (70%, BP 600/50; 400 ms, confocal pinhole of 25 μm), 488 nm (10%, BP 525/50; 200 ms, confocal pinhole of 25 μm), 405 nm (20%, BP 450/50; 200 ms, confocal pinhole of 25 μm). Stacks of 100 to 150 μm in size were acquired with a step size of 0.2 μm while acquiring all *Z* positions for each channel. At least five different regions were acquired for each condition. All scans were 2048 pixels by 2048 pixels, 16-bit, binning 1 by 1 with a frame averaging of 1. Images were deconvolved as reported above.

#### Morphology and paxillin-mCherry analysis

Deconvolved images were processed in Fiji by adjusting the minimum and maximum sliders of each channel for presentation purposes. No other image processing was performed.

#### Collagen remodeling experiments

For the images showing the remodeling of collagen with PIC-A488, cells were embedded in PIC(S) Alexa Fluor 488 at 0.2 mg ml^−1^ (1X PBS). After fixation, cells were stained with Alexa Fluor 647 phalloidin (1:250; A22287, Invitrogen), DAPI (1:10,000; D1306, Invitrogen), and recombinant mScarlet-I CNA35 collagen I–binding peptide (1:500) (*45*) in 1X PBS. Images were acquired in an inverted LSM880 Fast Airyscan (Zeiss) using a Plan Apochromat 40× water objective (1.2 N.A.) using GenTeal gel. Acquisition was performed sequentially in the following order 633 nm (BP 605/70), 561 nm (BP 605/70), 488 nm (BP 525/50), and 405 nm (BP 445/50) using a 3.0% laser power with a gain of 800% and digital gain of 1.0 for all channels. Scans were 1052 μm by 1052 μm (16-bit) and acquired in a bidirectional scan direction with a speed of 6 and line averaging of 1. Stacks of 100 to 150 μm were acquired using a 0.22-μm step size while acquiring all *Z* positions for each channel. Airy scan images were processed in the ZEISS software (Zeiss Zen 2012 Black). For the images showing the remodeling of collagen in reflection mode, telomerase-immortalized fibroblasts (TIFs) parentals were embedded in collagen and PIC-collagen composites for 24 hours before fixation. Cells were stained with Alexa Fluor 488 phalloidin (1:500) and DAPI (1:10,000, D1306, Invitrogen) in 1X PBS as reported. Images were acquired in an inverted confocal microscope (TCS SP8 WILL, Leica Microsystems) using HC PL APO CS2 40×/1.10 water immersion objective (Leica Microsystems). Collagen deformation was quantified with a coherency analysis (*49*). Fiber orientation and coherency of collagen fibers (defined as the co-orientation of collagen fibers pointing in the same direction) was measured by using Fiji plugin, OrientationJ. The coherency ratio was measured by selecting a pair of 15 μm–by–15 μm regions of interest (ROIs). One ROI on the axis of the cell protrusion and another one in the orthogonal position.

#### Bead experiments

Parental TIF cells were embedded in PIC-collagen composites containing fluorescent beads (Invitrogen TetraSpeck Microspheres, 1.0 μm, fluorescent blue/green/orange/dark red; Invitrogen, T7282) at a final bead concentration of 1:30 and imaged after 24 hours. Imaging was performed in a Nikon Ti-E inverted microscope equipped with a Hammamatsu Flash 4.0 sCMOS camera and controlled by NIS Elements software (Nikon) using a 20× dry objective (Plan Apochromat VC, 1.10 N.A.). Stacks of ~50 μm size were acquired with a step size of 5 μm (11 steps) while acquiring all *Z* positions for each fluorescent (488 nm) and bright-field channel. Images were acquired every 10 min for 24 hours. Beads were detected by size and tracked by using a simple linear assignment problem (LAP) tracker method in the TrackMate plugin in Fiji (*78*) and filtered by track speed to remove unattached beads. The position of the cell center was obtained by the bright-field channel in MATLAB. The maximal bead displacements were calculated for all beads within a 30-μm radius from the cell, as the maximal distance between two consecutive positions. The peaks of the bead displacement were obtained using MATLAB function findpeaks. Only peaks that exceed a value of 2 times the SD were included. The peak value and the width were used to calculate the slope as: peak/(0.5 × width).

### Software and statistical analysis

Statistical significance was determined in GraphPad Prism (version 10.0.2) for sets of data in Figs. 4 to 6 by first identifying outliner using the ROUT (*Q* = 1%) method and applying a normal (Gaussian) distribution test using a Shapiro-Wilk test distribution. We applied a nonparametric test using a Kruskal-Wallis one-way test and Dunn’s multiple comparison tests to assess significance. Data reports confidence level of 0.05. For bead experiments, beads were tracked by using a simple LAP tracker method in the TrackMate plugin in Fiji, and peaks of the bead displacement were obtained by custom scripts in MATLAB (MATLAB version R2021a). Analysis first by a D’Agostino-Pearson omnibus normality test then with ordinary one-way ANOVA with Dunnett’s multiple comparisons. Analysis and graphs include outliers identified. Adobe Illustrator 2022 (Adobe) was used to generate all figures.
