# Supplementary material for: Tuning collagen nonlinear mechanics with interpenetrating networks drives adaptive cellular phenotypes in three dimensions
Source: Sci Adv. 2025 Jun 20;11(25):eadt3352. doi: 10.1126/sciadv.adt3352 (PMC12180499; doi:10.1126/sciadv.adt3352)
Supplement: Supplementary file 1 — Figs. S1 to S8 Legends for movies S1 to S8 [file sciadv.adt3352_sm.pdf]

Science Advances
AAAS

Supplementary Materials for

Tuning collagen nonlinear mechanics with interpenetrating networks drives adaptive cellular phenotypes in three dimensions

Marco A. Enriquez Martinez *et al.*

Corresponding author: Marco A. Enriquez Martinez, m.enriquez@uq.edu.au; Samantha J. Stehbens, s.stehbens@uq.edu.au; Alan E. Rowan, alan.rowan@uq.edu.au

Sci. Adv. **11**, eadt3352 (2025)
DOI: 10.1126/sciadv.adt3352

The PDF file includes:

Figs. S1 to S8
Legends for movies S1 to S8

Other Supplementary Material for this manuscript includes the following:

Movies S1 to S8

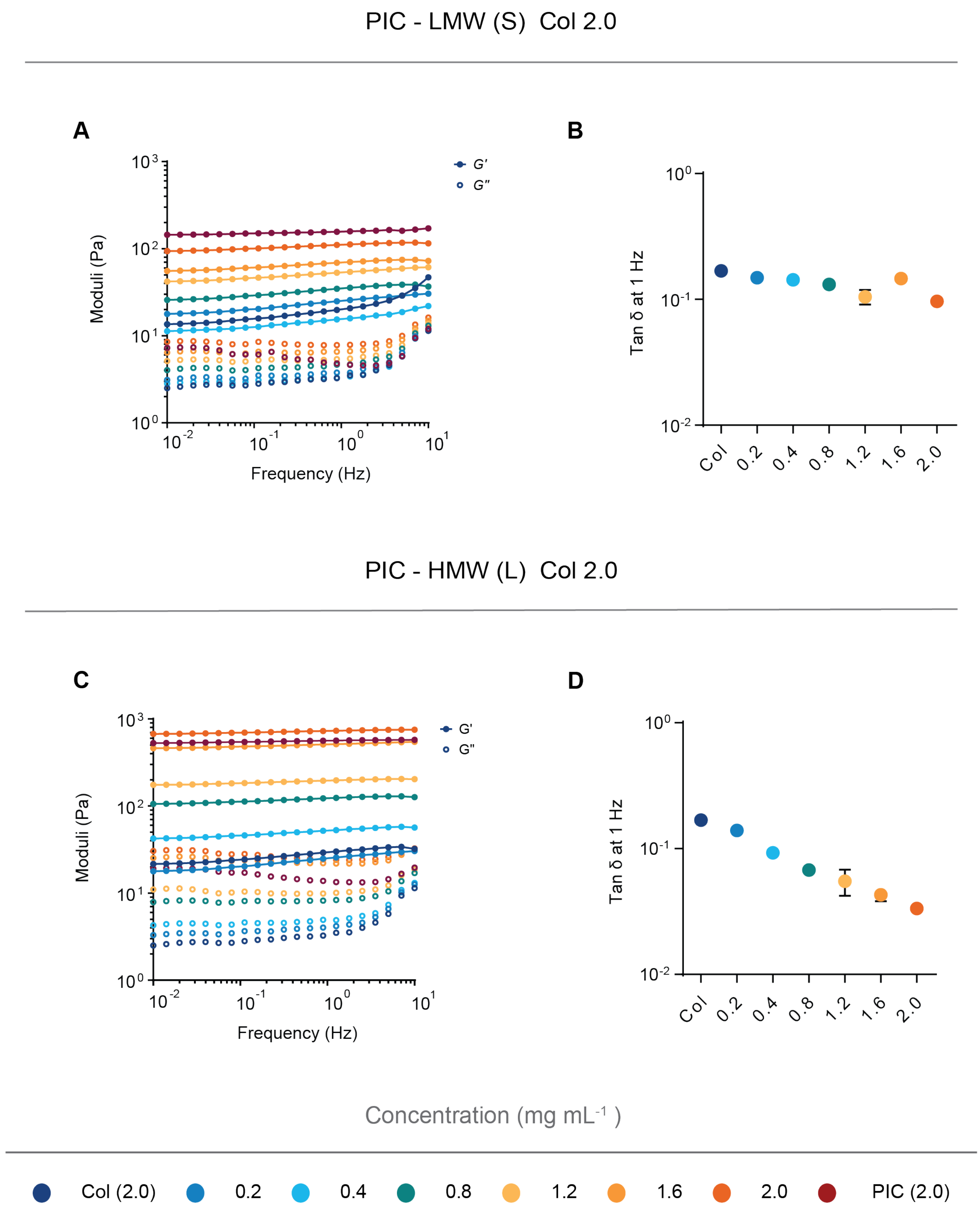

PIC - LMW (S) Col 2.0
A
B
Moduli (Pa)
Frequency (Hz)
G'
G"
Tan δ at 1 Hz
Col
0.2
0.4
0.8
1.2
1.6
2.0
PIC - HMW (L) Col 2.0
C
D
Moduli (Pa)
Frequency (Hz)
G'
G"
Tan δ at 1 Hz
Concentration (mg mL-1)
Col (2.0)
0.2
0.4
0.8
1.2
1.6
2.0
PIC (2.0)

Fig. S1. Extended linear mechanical characterisation of PIC-Collagen composites.

(**A-D**) The viscoelastic properties of the composite hydrogels were evaluated after gelation with applied frequency sweeps (10 - 0.01 Hz). (**B**,**D)** The *tan δ* (G''/G') profiles indicate that the viscoelastic profile of bovine atelo collagen is consistent throughout all the PIC-Collagen composites containing lower molecular weight (**A**,**B**), but the elasticity of the materials increases with an increase in PIC contour length (**C**,**D**). All sets of data represent three-independent measurements (n=3).

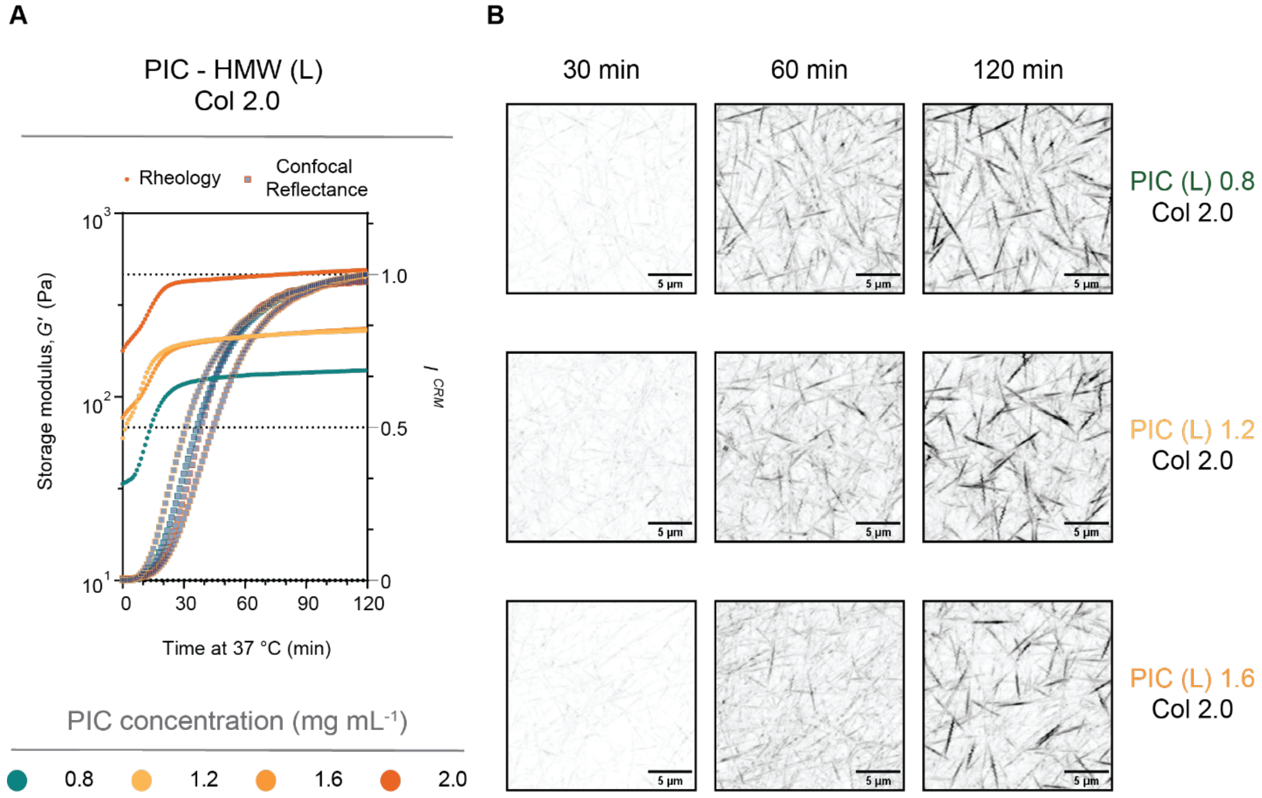

Fig. S2. Monitoring collagen fibrillogenesis with increasing PIC density using confocal rheology.

(**A-B**) The sol–gel transition of the PIC-Collagen hybrids occurs before the increase in I^{CRM}. Interestingly, all composites show an identical I^{CRM} profile indicating that the intensity profile collagen network formation is not accelerated with increasing PIC polymer density (**A**). This can also be observed by inverted confocal images (**B**). Z-projections show 20 µm depth. Scale bar (5 µm). Data represents an average of two independently prepared samples for each condition (n=2).

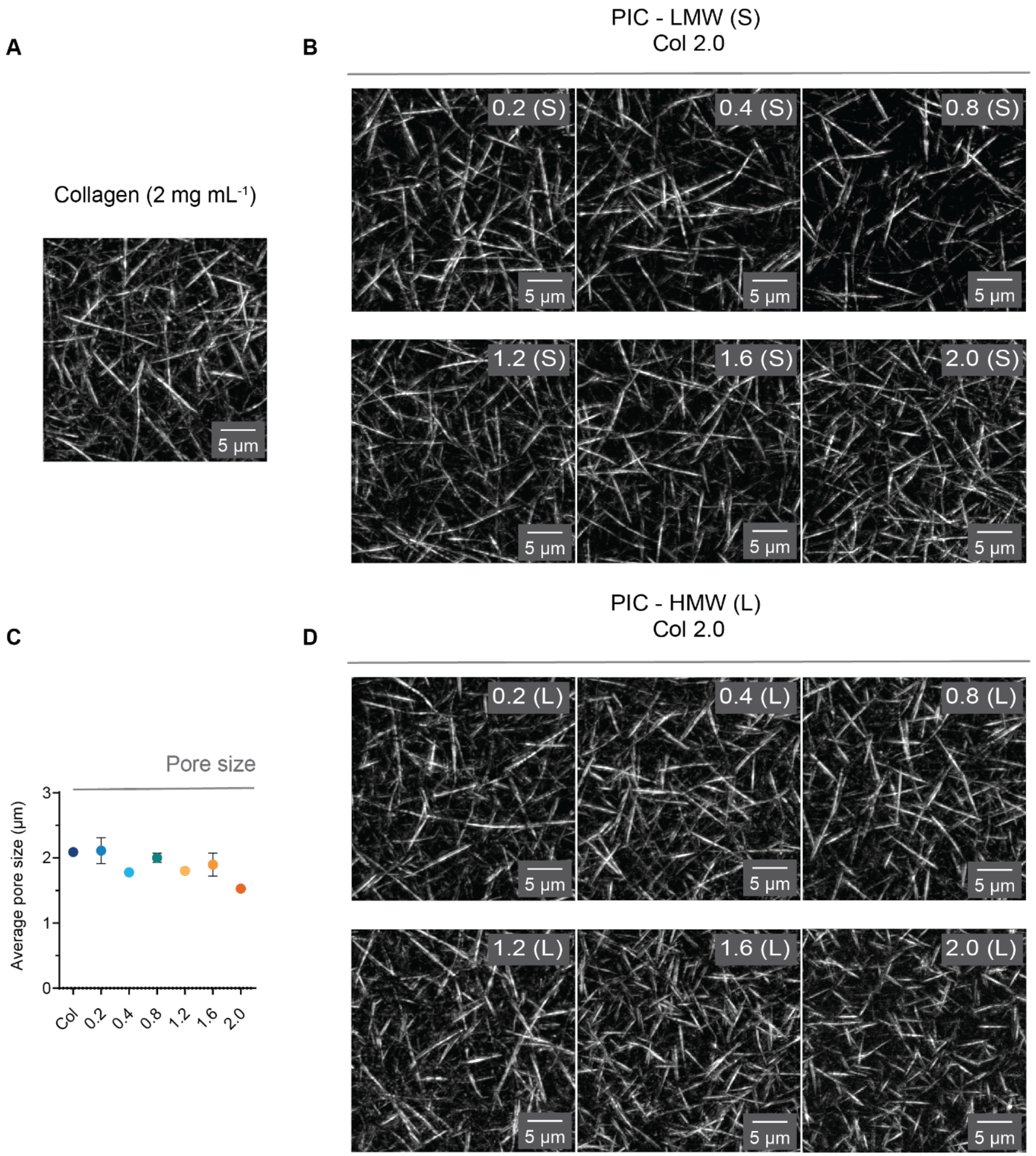

Fig. S3. Pore size analysis by confocal laser scanning microscopy.

Z-projections of representative confocal reflection images of (**A**) bovine atelo collagen (2 mg mL^{-1}) and (**B**) composites with increasing PIC concentrations (0.2–2.0 mg mL^{-1}) using LMW PIC and (**C-D**) using HMW PIC. Z-projections are 10 µm in depth. Scale bar (5 µm). (**C**) Quantitative bubble analysis indicates no considerable change in the average pore size of bovine atelo collagen with increasing HMW PIC. Analysis shows data of three regions (z-projection of 50 µm depth each) taken subsequently within each hydrogel containing HMW PIC indicating homogeneity between the networks throughout the samples. Data represents an average of at least two independent measurements (n=2).

Table 1. Individual parameters of stress-stiffening response of composites using low molecular weight PIC.

LMW PIC (mg mL^{-1})	Atelo Collagen (mg mL^{-1})	G_0 (Pa)	σ_c (Pa)	m
-	2.0	23.1 ± 3.8	0.35 ± 0.02	1.1
0.2	2.0	27.2 ± 2.6	0.59 ± 0.12	1.2
0.4	2.0	27.5 ± 3.2	0.65 ± 0.10	1.2
0.8	2.0	38.2 ± 5.0	0.91 ± 0.07	1.1
1.2	2.0	54.6 ± 20.5	1.63 ± 0.39	1.0
1.6	2.0	68.0 ± 25.7	2.16 ± 0.65	1.1
2.0	2.0	109.4 ± 41.7	4.21 ± 1.41	1.1
2.0	-	156.1 ± 14.8	11.81 ± 2.20	1.5

Table 2. Individual parameters of stress-stiffening response of composites using high molecular weight PIC.

HMW PIC (mg mL^{-1})	Atelo Collagen (mg mL^{-1})	G_0 (Pa)	σ_c (Pa)	m
-	2.0	23.1 ± 3.8	0.35 ± 0.02	1.1
0.2	2.0	33.2 ± 0.7	0.64 ± 0.04	1.1
0.4	2.0	51.7 ± 1.6	1.69 ± 0.15	1.1
0.8	2.0	122.3 ± 30.7	4.03 ± 0.95	1.0
1.2	2.0	194.4 ± 97.4	10.20 ± 7.73	1.1
1.6	2.0	523.3 ± 57.8	22.50 ± 4.54	1.1
2.0	2.0	748.8 ± 140.9	31.41 ± 7.68	1.0
2.0	-	487.2 ± 29.4	30.05 ± 3.35	1.5

Fig. S4. Extended Individual parameters of stress-stiffening response of collagen composites with LMW and HMW PIC.
All sets of data represent three-independent measurements (n=3).

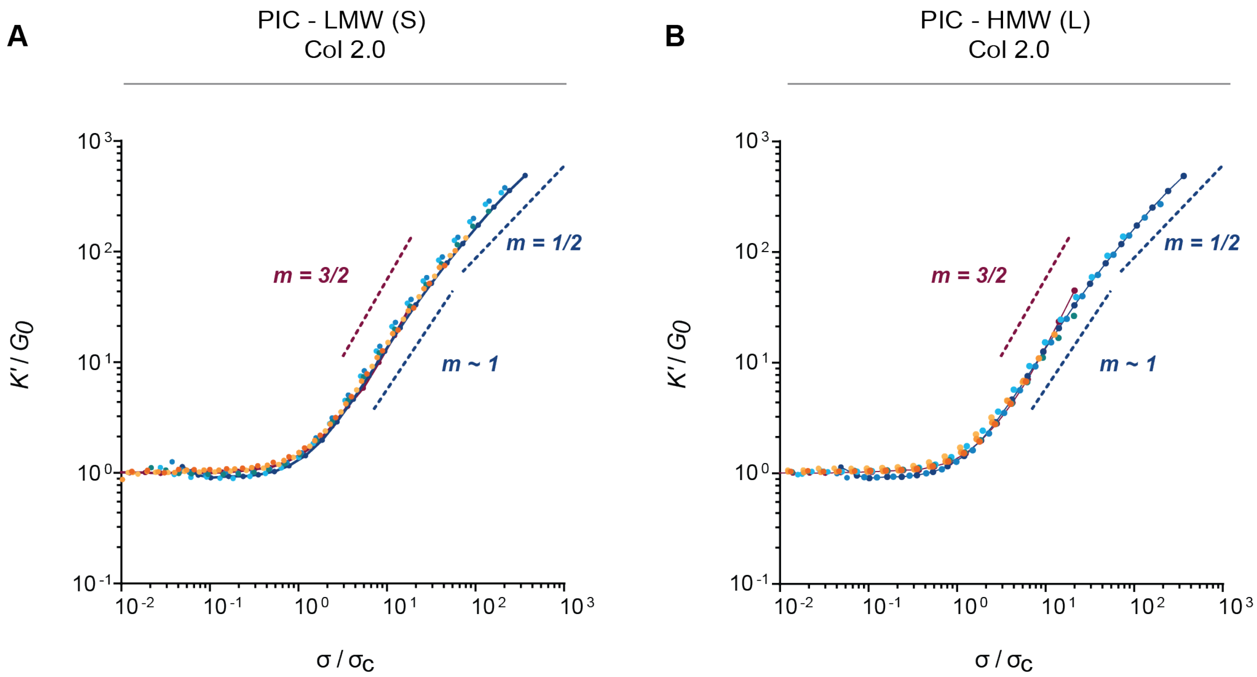

Fig. S5. Fiber bending interactions dominate the non-linear response of PIC-Collagen composites.

(**A, B**) Single master curves of PIC-Collagen composites were generated by scaling the differential modulus (K') with the linear modulus (G_0) and σ with σ_c. The differential modulus (K') of the PIC-Collagen composites follows $K' \propto \sigma^1$ characteristic of initial fiber bending of collagen network in the non-linear regime. Note that as the concentration of PIC increases, K' doesn't follow an entropic stiffening where $K' \propto \sigma^{3/2}$.

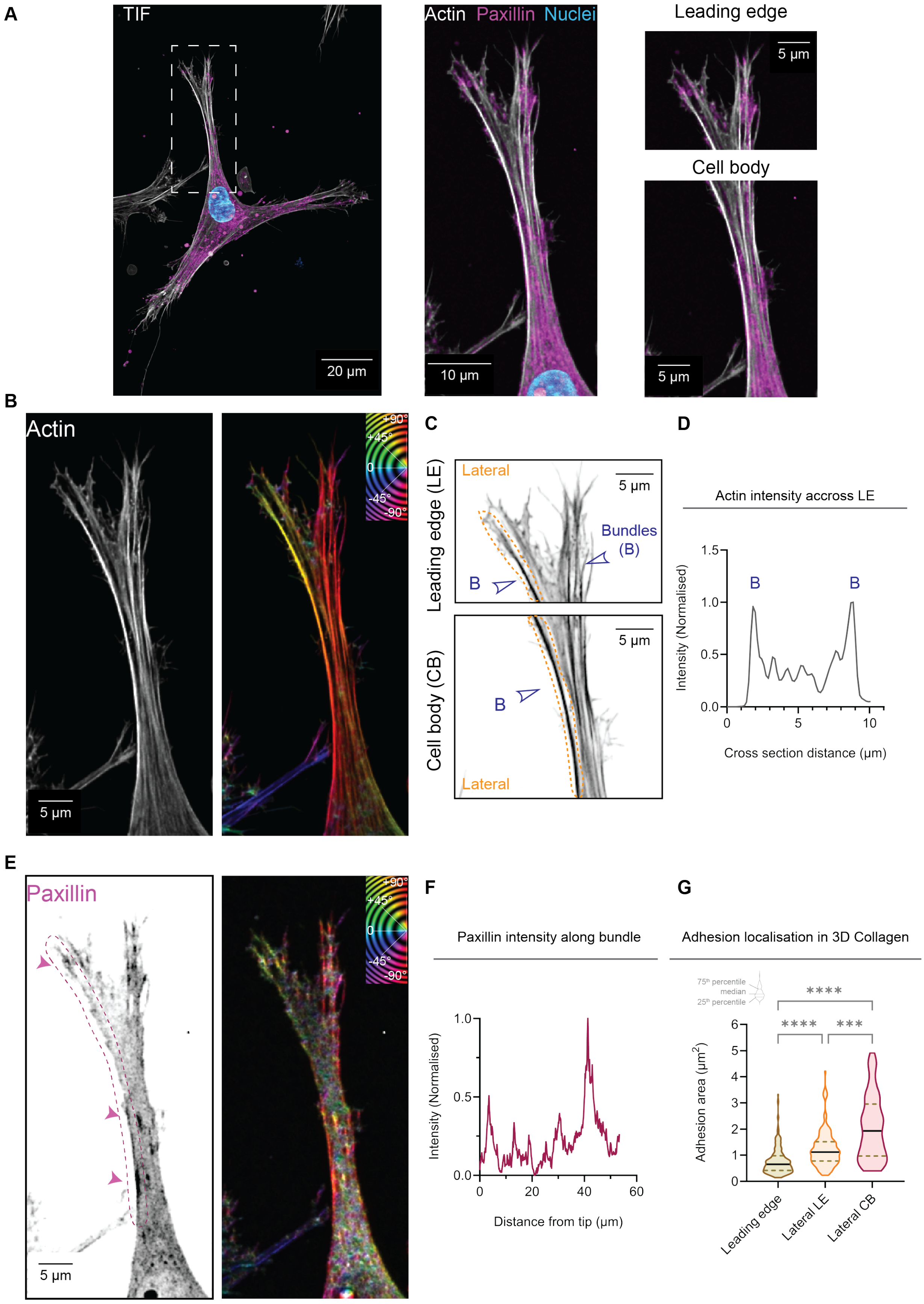
A
TIF
20 µm
Actin Paxillin Nuclei
10 µm
Leading edge
5 µm
Cell body
5 µm
B
Actin
5 µm
+90°
+45°
0
-45°
-90°
C
Leading edge (LE)
Lateral
5 µm
Bundles (B)
B
Cell body (CB)
5 µm
B
Lateral
D
Actin intensity accross LE
Intensity (Normalised)
Cross section distance (µm)
E
Paxillin
5 µm
F
Paxillin intensity along bundle
Intensity (Normalised)
Distance from tip (µm)
G
Adhesion localisation in 3D Collagen
75th percentile
median
25th percentile
****
****
***
Adhesion area (µm²)
Leading edge
Lateral LE
Lateral CB

Fig. S6. Spatial organisation of focal adhesions and the actin cytoskeleton in fibroblast cells embedded in 3D collagen hydrogels.

(**A**) Confocal image of a fixed fibroblast expressing Paxillin–mCherry embedded in an atelo bovine collagen hydrogel (2 mg mL^{-1}, 1X PBS, pH 7) after 24 hours. Actin and nuclei are stained with Phalloidin 488 and DAPI. Z projection ~50 µm. Scale bar (20 µm). Inlet shows a zoomed region of a cell extension of interest (Scale bar (10 µm)) and the extension is classified into two subregions: leading edge and cell body. Scale bar (5 µm). (**B**) Organisation of the actin cytoskeleton at leading edge and cell body is depicted by the colour coded orientation (°, degrees) map of actin bundles and (**C**) inverted confocal images**.** Both arrows and (**D**) intensity profile (normalised) of a straight-line scan drawn across the body indicate an accumulation of F-actin localised at the lateral sides of both leading edge and cell body. Scale bar (5 µm). (**E**) Localisation of paxillin at both leading edge and lateral sides of leading edge/cell body shown by colour coded orientation of adhesions (paxillin) (°, degrees) and inverted confocal images. Note the matching colour coding of the adhesions and actin structures indicating an alignment and orientation of adhesions relative to the bundles. (**F**) Intensity profile (normalised) of a line scan drawn from tip to cell body (~60 µm) following the direction of the actin bundle indicates different morphological phases of adhesions along the cell extension. (**G**) Adhesions at leading edge are smaller compared to adhesions at the lateral regions of the leading edge and cell body. Adhesions were manually quantified (Leading edge (n= 213), Lateral leading edge (n = 168), Lateral cell body (n = 69)) from a set of two independent measurements (30 cell extensions from 14 cells). Violin plots (truncated, medium smooth) show median (black line) and quartiles (gold pattern lines).

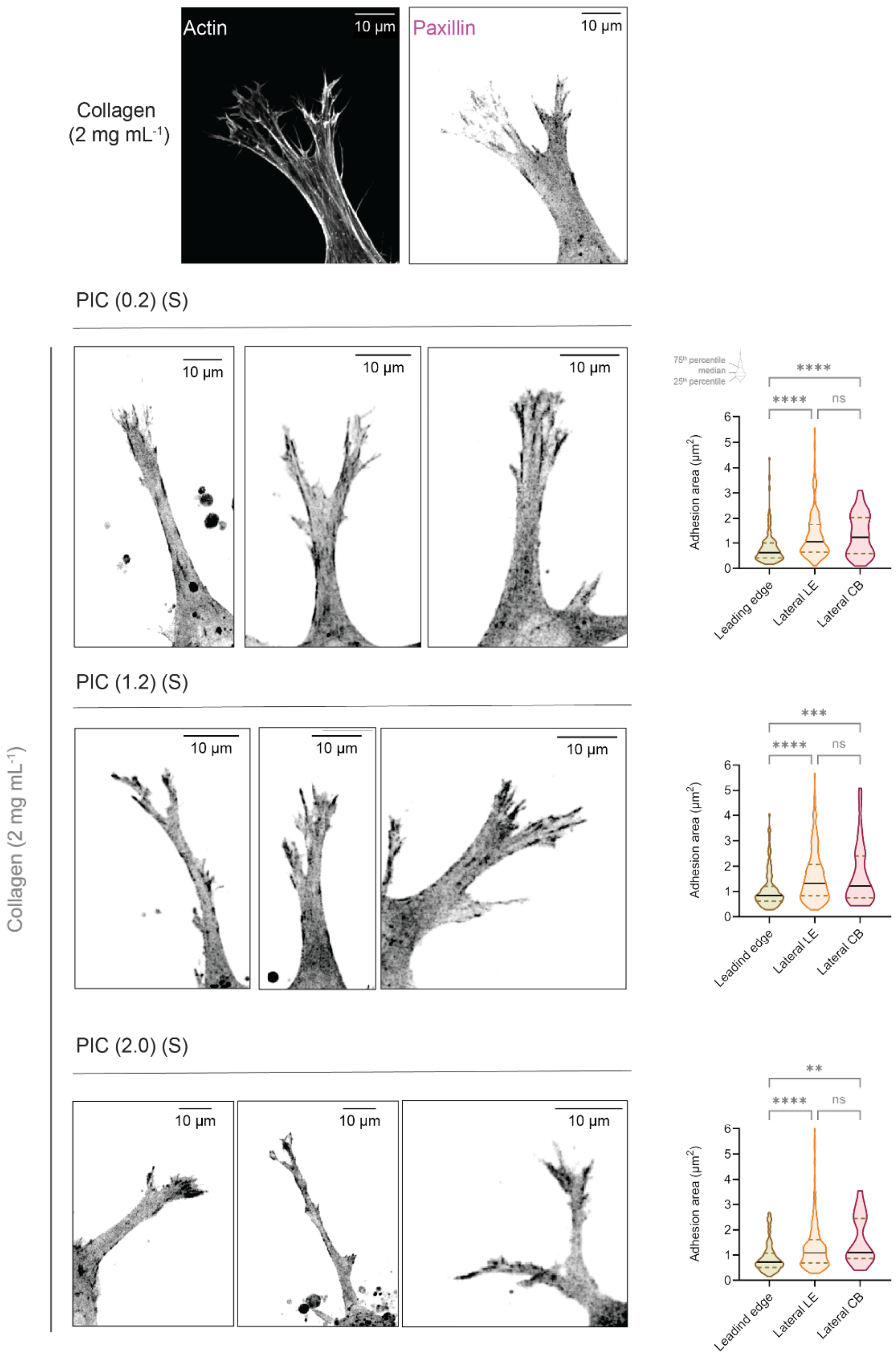

Fig. S7. Extended data of cell-matrix interactions in PIC-Collagen composites.

Representative confocal images showing the localisation of paxillin at both leading edge and lateral sides of leading edge/cell body. In comparison to the localisation in pure collagen hydrogels, adhesions at the leading edge are smaller compared to adhesions at the lateral regions of the leading edge and cell body. Violin plots (truncated, medium smooth) show median (black line) and quartiles (gold pattern lines).

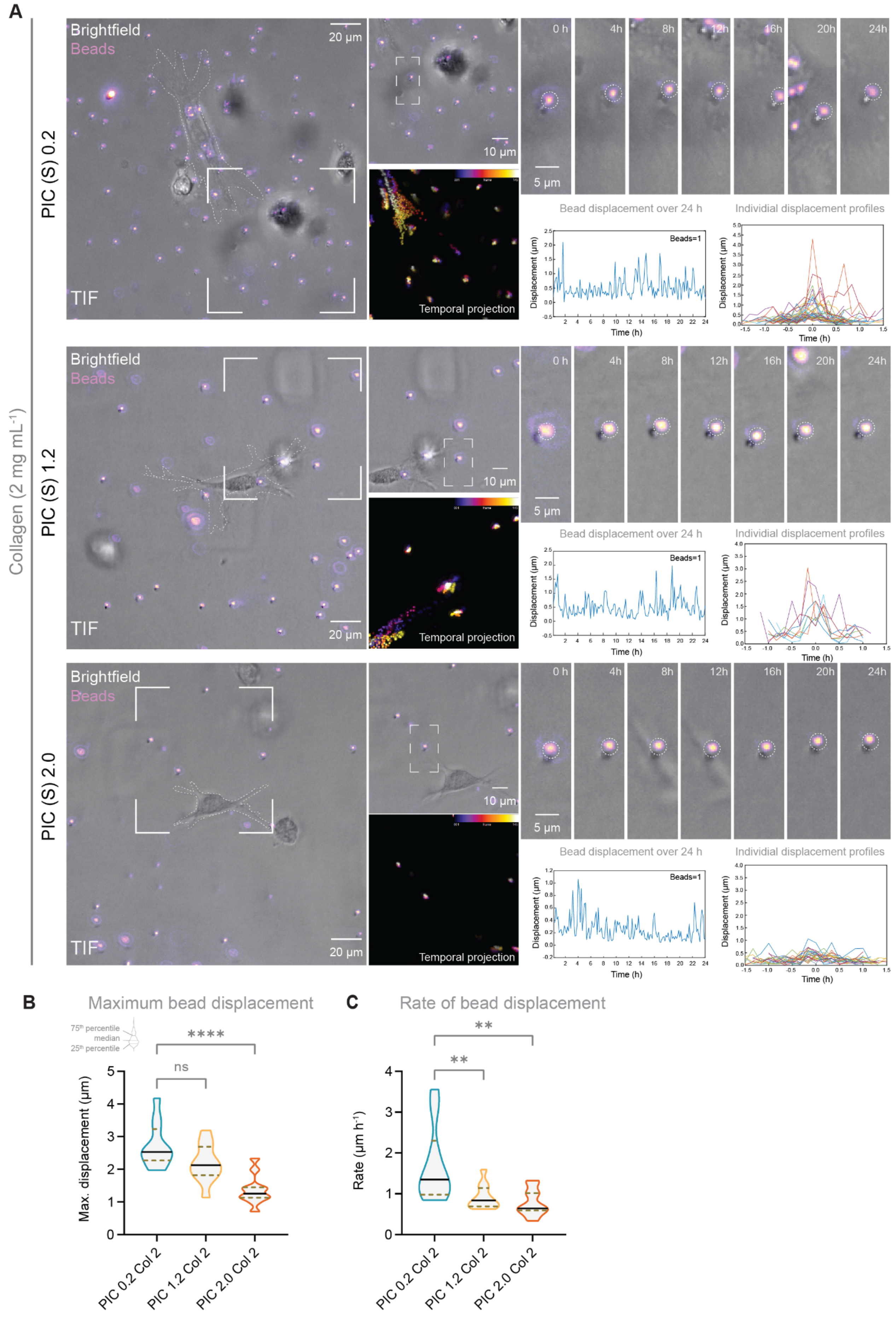
A
Collagen (2 mg mL-1)
PIC (S) 0.2
PIC (S) 1.2
PIC (S) 2.0
Brightfield
Beads
TIF
20 µm
10 µm
5 µm
Temporal projection
0 h
4h
8h
12h
16h
20h
24h
Bead displacement over 24 h
Individial displacement profiles
Beads=1
Displacement (µm)
Time (h)
B
Maximum bead displacement
75th percentile
median
25th percentile
****
ns
Max. displacement (µm)
PIC 0.2 Col 2
PIC 1.2 Col 2
PIC 2.0 Col 2
C
Rate of bead displacement
**
**
Rate (µm h-1)

Fig. S8. Bead displacements by fibroblasts in PIC-Collagen composites.

(**A**) Representative time-lapses of fluorescent microbeads (TetraSpeck^{TM} microspheres,1 µm) being displaced by fibroblasts embedded in PIC-Collagen composites (LMW) (0.2, 1.2, 2.0 mg mL^{-1}) after 24 hours. Images were acquired every 10 min for 24 hours. Time is shown in hours (h). Scale bar (20 µm). Z projections are ~10 µm depth (Z=2 steps). Insets show individual fluorescent beads surrounding cellular extensions. Scale bar 10 µm and 5 µm, respectively. Graph (left) shows an example of the displacements applied by fibroblasts on a single bead over 24 hours (blue line). Bead displacements were calculated from the distance between two consecutive positions from trajectories obtained with TrackMate plugin in FIJI. Graph (right) shows the individual centered peaks detected from all beads surrounding a single cell (coloured lines) that were used to calculate the rate of displacement (µm h^{-1}). Rate of displacement was obtained from the maximum displacement value divided by half of the peak's width. (**B**) Maximum bead displacement (µm) (PIC 0.2 Col 2, n=76 beads from 11 cells; PIC 1.2 Col 2, n=75 beads from 11 cells; PIC 2.0 Col 2, n=77 beads from 11 cells). (**C**) Rate of bead displacement (µm h^{-1}) (PIC 0.2 Col 2 n=364 peaks from 11 cells; PIC 1.2 Col 2 n=339 peaks from 11 cells; PIC 2.0 Col2 n=392 peaks from 11 cells). For all sets of data, data was first analysed for normality using a D'Agostino-Pearson omnibus normality test then analysed with ordinary one-way ANOVA with Dunnett's multiple comparisons with PIC 0.2 Col 2 as reference. For all the statistical analysis, ns, $P > 0.05$; ** $P < 0.01$; **** $P < 0.0001$. Violin plots (truncated, medium smooth) show median (black line) and quartiles (gold pattern lines).

Movie S1. Collagen self-assembly monitored by confocal rheology.

Time-lapse imaging showing the formation of atelo collagen networks (2 mg mL^{-1}, bovine atelo, 1X PBS, pH 7) using confocal reflection microscopy. Confocal images were acquired using a combined laser scanning microscope with a rheometer. Confocal images were acquired with a 40x water objective (HC PL APO CS2, 1.10 N.A.) every 1 min while applying a temperature ramp from 5 – 37 °C for 30 min (0:00-0:30 min) (1 °C min^{-1}) and a constant temperature of 37 °C for 2 hours (00:30 min – 2 h:30 min). Inverted images are shown as a maximal projection of 20 µm depth. Scale bar 10 µm.

Movie S2. Sequential formation of PIC-Collagen IPN.

Time-lapse imaging showing the rapid assembly of the PIC network (orange) (0.8 mg mL^{-1} of azide-functionalised PIC (1/100) with Alexa Fluor ® 488; A488) followed by the formation of the collagen network (cyan) (2 mg mL^{-1}, bovine atelo), indicating that the PIC-Collagen system follows a sequential IPN formation. Confocal images were acquired using a combined laser scanning microscope with a rheometer. Confocal images were acquired with a 40x water objective (HC PL APO CS2, 1.10 N.A.) every 1 min while applying a temperature ramp from 5 – 37 °C for 30 min (0:00-0:30 min) (1 °C min^{-1}) and a constant temperature of 37 °C for 2 hours (00:30 min – 2 h:30 min). Reflection images were acquired between stacks in the first line with a 650 nm laser followed by fluorescence images with a 488 nm laser. Confocal images are shown as a maximal projection of 10 µm depth. Scale bar 10 µm.

Movie S3. Three-dimensional reconstruction of PIC-Collagen IPN.

Three-dimensional reconstruction of the PIC-Collagen IPN (0.8 mg mL^{-1} PIC(LMW)-A488; 2 mg mL^{-1} collagen) showing an interpenetration between networks. The confocal image was acquired using a combined laser scanning microscope with a rheometer to show a fully assembled PIC network at 37 °C. X-Y-Z stack shows 60 µm x 60 µm x 20 µm depth.

Movie S4. 1205 Lu melanoma in PIC (LMW)-Collagen IPNs.

Time lapse spinning-disc live-cell imaging of 1205 Lu melanoma expressing H2B-mScarlet-I after being embedded in PIC-Collagen (0.2, 0.8, 1.2 and 2 mg mL^{-1} LMW PIC; 2 mg mL^{-1} atelo bovine collagen) composites for 72 hours. Brightfield and confocal images were acquired with a 20x dry objective (Plan Apo VC, 0.75 N.A.) every 10 min for 12 hours while maintaining a temperature of 37 °C and 5% CO_2.

Movie S5. 1205 Lu melanoma in PIC (HMW)-Collagen IPNs.

Time lapse spinning-disc live-cell imaging of 1205 Lu melanoma expressing H2B-mScarlet-I after being embedded in PIC-Collagen (0.2, 0.8, 1.2 and 2 mg mL^{-1} HMW PIC; 2 mg mL^{-1} atelo bovine collagen) composites for 72 hours. Brightfield and confocal images were acquired with a 20x dry objective (Plan Apo VC, 0.75 N.A.) every 10 min for 12 hours while maintaining a temperature of 37 °C and 5% CO_2.

Movie S6. TIFs in Collagen and PIC-Collagen IPNs.

Time lapse spinning-disc live-cell imaging of telomerased-immortalised fibroblasts (TIF) expressing Paxillin-mCherry (fire) showing remodelling of surrounding collagen (CNA35-eGFP) (gray) in collagen-only hydrogels (Collagen 2mg mL-1; atelo bovine) and PIC-Collagen IPNs (0.2 and 1.2 mg mL^{-1} LMW PIC; Collagen 2 mg mL^{-1}, atelo bovine). Confocal images were acquired with a 25x silicone objective (CFI Plan Apochromat Lambda S,1.05 N.A.) sequentially between stacks of 40 µm (2 µm step; 21 steps) for 3 hours every 3 minutes while maintaining a temperature of 37 °C and 5% CO_2.

Movie S7. Collagen remodelling by TIFs during the formation of cellular extensions.

Time lapse spinning-disc live-cell imaging of telomerased-immortalised fibroblasts (TIF) expressing Paxillin-mCherry (magenta) in collagen-only hydrogels (Collagen 2mg mL-1; atelo bovine). Insets shows the deformation of the surrounding collagen (gray) (CNA35-eGFP) during the formation of cellular extensions. Confocal images were acquired with a 25x silicone objective (CFI Plan Apochromat Lambda S,1.05 N.A.) sequentially between stacks of 40 µm (2 µm step; 21 steps) for 3 hours every 3 minutes while maintaining a temperature of 37 °C and 5% CO_2.

Movie S8. Bead displacements by TIFs in PIC-Collagen IPNs.

Time lapse widefield live-cell imaging of telomerased-immortalised fibroblasts (TIF) embedded in PIC-Collagen IPNs (0.2, 1.2 and 2.0 mg mL^{-1} LMW PIC; Collagen 2 mg mL^{-1}, atelo bovine) after 24 hours. Widefield images were acquired with a 20x dry objective (Plan Apochromat VC, 1.10 N.A.) sequentially between stacks of ~50 µm (5 µm step; 11 steps) for 24 hours every 10 minutes while maintaining a temperature of 37 °C and 5% CO_2. Time lapses show maximal projections of 10 µm depth only.
